# Prevalence of dementia among older age people and variation across different sociodemographic characteristics: a cross-sectional study in Bangladesh

**DOI:** 10.1016/j.lansea.2023.100257

**Published:** 2023-08-24

**Authors:** Aliya Naheed, Maliha Hakim, Md Saimul Islam, Md Badrul Islam, Eugene Y.H. Tang, Abdul Alim Prodhan, Mohammad Robed Amin, Blossom C.M. Stephan, Quazi Deen Mohammad

**Affiliations:** a Initiative for Non Communicable Diseases, Health Systems and Population Studies Division, icddr,b, Mohakhali, Dhaka, 1000, Bangladesh; b National Institute of Neurosciences & Hospital, Dhaka, 1207, Bangladesh; c Laboratory Science and Services Division, icddr,b, Mohakhali, Dhaka, 1000, Bangladesh; d Population Health Sciences Institute, Newcastle University, UK; e Non Communicable Disease Control Program, Directorate General of Health Services, Dhaka, 1212, Bangladesh; f Department of Medicine, Dhaka Medical College and Hospital, Dhaka, 1000, Bangladesh; g Institute of Mental Health, Mental Health and Clinical Neurosciences, School of Medicine, University of Nottingham, Nottingham, UK; h Dementia Centre of Excellence, Curtin enAble Institute, Curtin University, Perth, Western Australia, Australia

**Keywords:** Dementia, Prevalence, Older age, Bangladesh

## Abstract

**Background:**

Dementia is a significant global health issue, particularly for low-income and middle-income countries which majorly contribute to the dementia cases reported globally (67%). We estimated the prevalence of dementia among older people in Bangladesh and compared the estimate across different sociodemographic characteristics and divisions.

**Methods:**

A cross-sectional study was conducted in 2019 among individuals aged 60 years or older in seven administrative divisions in Bangladesh. Equal numbers of male and female participants were recruited from each division through a multi-stage random sampling technique. Recruitment was proportionally distributed in urban and rural areas in each division. Following consent, the Mini Mental State Examination (MMSE) was performed on all participants. Dementia was defined as an MMSE score of <24 out of 30. Data on age, sex, education, marital status, occupation, socioeconomic status, and type of community (urban or rural) were obtained using a structured questionnaire to compare the prevalence of dementia across different sociodemographic characteristics.

**Findings:**

Between January and December 2019, 2795 individuals were recruited including ∼400 from each of the seven administrative divisions. The mean age was 67 years (SD: 7), 68% were from rural areas and 51% were female. The prevalence of dementia was 8.0% (95% CI: 7.0–8.9%) with variations across age, sex, education, marital status, occupation, and division. No variations in prevalence were observed across urban/rural locations or socioeconomic status. After adjusting for age, sex, education, occupation and marital status, the odds of dementia was two times higher in females than males (OR: 2.15, 95% CI: 1.43–3.28); nine times higher in people aged ≥90 years than people aged 60–69 years (OR: 9.62, 95% CI: 4.79–19.13), and three times higher in people with no education compared to those who had completed primary school (OR: 3.10, 95% CI: 1.95–5.17).

**Interpretations:**

The prevalence of dementia is high in Bangladesh and varies across sociodemographic characteristics with a higher prevalence among females, older people, and people with no education. There is an urgent need to identify the key risk factors for dementia in developing countries, such as Bangladesh, to inform the development of context-relevant risk reduction and prevention strategies.

**Funding:**

None.

**Research in context**

**Evidence before this study**

The WHO estimated that in 2021 there were 57.6 million people with dementia worldwide with a disproportionate prevalence in low-income and middle-income countries (LMICs). However, compared to high-income countries (HICs), information on dementia is very fragmented and often non-existent in LMICs, including Bangladesh. We searched PubMed and Google Scholar until August 2022, for articles published in English, using the terms “dementia”, “cognitive impairment”, “cognition”, “sociodemographic characteristics” and “Bangladesh”. No studies were identified that have reported the country level prevalence of dementia and investigated variation in prevalence by sociodemographic characteristics or type of community (i.e., rural vs. urban).

**Added value of this study**

The current study shows findings from the first national dementia survey in Bangladesh. We project that the prevalence of dementia will almost triple in number over the next three decades. Sociodemographic risk factors for dementia were like those reported in HICs, including increased age, female sex, low educational attainment and being single (marital status), but not type of community (e.g., urban vs. rural). However, this study has demonstrated regional variation (i.e., across the administrative divisions of Bangladesh) in dementia prevalence as well as interactions between sex and basic sociodemographic characteristics, which have not been reported before in LMIC settings.

**Implications of all the available evidence**

Improving population health and reducing risk of dementia in Bangladesh would have a significant impact not only on the individuals themselves, but their family/community, health systems and healthcare budgets. New initiatives and policies that prioritise budgets and healthcare resources to identify key risk factors for dementia and instigate management, risk reduction and prevention are urgently needed. Any efforts must however consider socio-cultural factors, mental health stigma, and available infrastructure.

## Introduction

Dementia is a general term for significant decline in cognitive and physical functioning.^1^ Currently dementia affects 57.6 million people worldwide, with approximately 10 million new cases diagnosed each year.^2^ Most people (∼67%) with dementia live in the low-income and middle-income countries (LMICs), and by 2050, this estimate is projected to reach 152.8 million cases.^3^ As such, dementia is a major concern for LMICs as most do not have access to disease estimates or information on the pattern of key risk and protective factors. Further, most LMICs do not have policies in place to deal with the social and economic burdens of dementia, or programs of risk reduction necessary to impact future numbers.^4^^,^^5^

Recent findings on global temporal trends in dementia prevalence and incidence suggest that risk of dementia is declining in some high-income countries (HICs) (e.g., in Europe and the USA), while it is remaining stable or increasing in other countries.^6^, ^7^, ^8^ However, relative to HICs, there is less data on dementia in LMIC settings and it is not clear whether similar trends are being observed.^9^ Where prevalence estimates are available, they vary e.g., from 1% to 15% in South-East Asian countries and 2.3%–23% in African countries.^10^ The highest number of dementia cases is currently reported in Asia (22.9 million), which is more than double the number reported in Europe (10.5 million) or the Americas (9.4 million).^11^ There are approximately 4.8 million people with dementia in South Asia, including 3.8 million in India (0.3% of the total population), 0.6 million in Bangladesh (0.3% of the total population) and 0.4 million in Pakistan (0.2% of the total population).^12^ By 2050, due to population ageing, the number of dementia cases is expected to rise to 24.7 million in South Asia including 11.4 million in India (0.7% of the total population), 2.0 million in Bangladesh (0.8% of the total population) and 1.4 million in Pakistan (0.4% of the total population).^12^ Discrepancies in prevalence estimates across world regions is likely due to heterogeneity in study methodology (e.g., recruitment, dementia diagnostic criteria), age distribution of the study participants and differences in the profile of risk/protective factors across sites (e.g., level of educational attainment and socioeconomic status [SES]).^13^, ^14^, ^15^, ^16^

Currently, there is no national estimate of the prevalence of dementia in Bangladesh. A single community-based study conducted in 2003–2004 in a rural sub-district (Matlab, Chandpur) reported dementia prevalence to be 15.1%.^17^ However, this estimate only represents a small geographic area in Bangladesh and is not generalisable to the whole country. Thus, knowledge about the current and future number of dementia cases in Bangladesh, and any variation across different socio-demographic groups and regions is imperative for developing context-relevant strategies for risk reduction and prevention. Therefore, the aim of this study is to estimate, for the first time, the population prevalence of dementia among adults aged ≥60 years in Bangladesh, and compare the prevalence of dementia across age, sex, SES, type of community (i.e., rural vs. urban) and regions (administrative divisions).

## Methods

### Study site

**Fig. 1:**
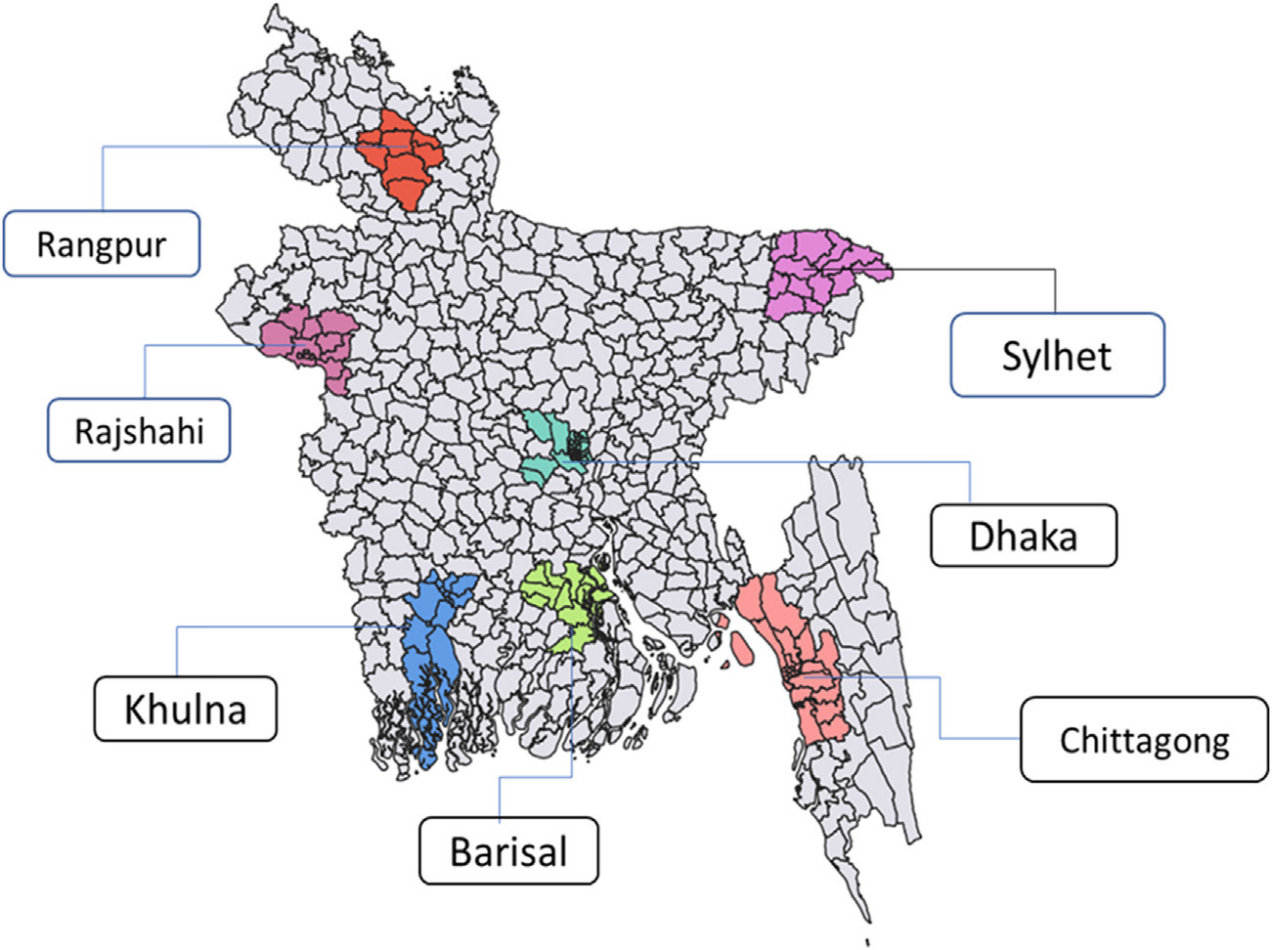
Selected study sites from seven administrative divisions of Bangladesh.

From January to December, 2019, we conducted a multistage cross-sectional study in urban and rural areas of seven administrative divisions in Bangladesh that had a fully functional City Corporation authority before 2018: Dhaka, Chattogram (Chittagong), Khulna, Barisal, Sylhet, Rajshahi, and Rangpur.^17^^,^^18^ A district in each division was selected that included a City Corporation representing the major urban area of that division (divisional district). A City Corporation includes several wards representing the lowest administrative urban units in a division. Much of a divisional district (apart from a City Corporation) includes several sub-districts representing the rural areas of a division. A sub-district includes several union parishad (UP) representing the lowest rural administrative units in a division. One urban ward was randomly chosen from a City Corporation area, and one rural UP was randomly chosen from a divisional sub-district in each of the seven divisions. In total, seven rural UPs and seven urban wards were selected for recruitment of study participants from each division (Fig. 1).

### Study participants

The sample size calculation was based on a cross-sectional study conducted in a rural sub-district of Bangladesh with an estimated dementia prevalence of 15.1%.^17^ Based on this estimate, we needed to recruit at least 196 participants per division assuming 95% level of significance, 5% margin of error and 10% non-response rate. As such, we aimed to recruit around 400 participants per division with a 1:1 male to female ratio and a total of 2800 participants from seven divisions. Based on a sample of 2800 participants, 15% prevalence and 95% level of significance, the expected Wald confidence interval of dementia prevalence would be 13.7%–16.3%. To inform recruitment we developed a sample frame of people aged ≥60 years by listing every member of each household included in the selected urban ward and rural UP in each division (Appendix 1, Figure S1). A simple random sampling technique, without replacement, was applied until the target sample size was achieved. Eligibility criteria included an age of ≥60 years, having resided in the community for at least six months, and able to provide written consent. If two or more eligible participants resided in the same household, one of them was recruited at random. Temporary residents (i.e., those living less than six months in the study area) and those who were too ill to participate were excluded from the study. We also excluded individuals who were deaf or blind, since hearing or vision impairment may affect an individual's ability to perform the Mini–Mental State Examination (MMSE).

### Data collection

Trained research assistants who had at least a college degree interviewed participants at their home. The interview schedule included an assessment of cognitive function using the MMSE (score range: 0–30). The MMSE was administered in Bangla language^19^, ^20^, ^21^ and has been validated in Bangladesh previously.^22^ A neurologist trained the research assistants to administer the MMSE and inter-observer reliability was assessed among the research staff prior to administration of the MMSE to study participants. Additional data were collected following administration of the MMSE that included information on age, sex, education, marital status, employment, and household assets in the presence of a caregiver or an adult family member.

### Dementia diagnosis

Any individual with an MMSE score of <24 was classified as having dementia.^23^ For cases where the MMSE score was borderline (i.e., MMSE = 24), the study doctor further evaluated the individual (within one week of interview) including re-administering the MMSE to determine dementia status.

### Data quality

Data quality was assured through training of the field staff, extensive supervision through spot visits by senior investigators in the field and re-interviewing 2% of the participants within 24 h. MS Access, with range and consistency built in checks, was used for data entry. Data cleaning was done as per the standard procedures of the research institute.^24^ All survey forms were reviewed by the team leader for necessary corrections and 5% of the participants were re-interviewed by a different group to cross-check the quality of data.

### Data analysis

A wealth index was created by applying principal component analysis to the household asset data to determine SES categorised as lower, lower-middle, middle, upper-middle, and upper as recommended by the Demography and Health Survey in 2020^25^ (For full details of the methods see Appendix 2). Summary measures are reported as mean and SD for continuous variables and as frequency distributions for categorical variables. Bivariate analyses were used to explore differences in sociodemographic characteristics including age, sex, marital status, education, SES, and occupation across the seven divisions of Bangladesh. The Kruskal–Wallis one-way ANOVA was used for non-normally distributed variables to compare differences in median age across the seven divisions.

The prevalence of dementia was estimated as the percentage of participants who had an MMSE score <24 out of the total number of participants recruited. The population level estimate of dementia was calculated as the number of individuals with an MMSE <24 per 100,000 older people. The projected prevalence of dementia was calculated by multiplying the prevalence of dementia reported in the current study with the total number of older age people projected for 2025, 2041, and 2051 (after 3 years, 19 years, and 29 years respectively), based on the Census Report of Bangladesh 2011.^26^

Bivariate analyses were conducted to compare the prevalence of dementia by age (at every 10-year interval), sex (male or female), years of formal education (never went to school was defined as ‘no education’, 1–4 years of schooling was defined as ‘some education’ and ≥5 years of schooling was defined as ‘completed primary’), marital status (married or single), employment level (employed or unemployed), community type (urban or rural), administrative division and SES (i.e., wealth status). Independent sample t-tests were used for continuous variables (normally distributed) and the Chi-square test for categorical variables in bivariate analyses. Point biserial correlation (pbc) was calculated between the prevalence of dementia and age in years and years of education.^27^ Univariate logistic regression models were run to examine the association between dementia prevalence and each sociodemographic characteristic, separately.

We also ran a multivariable model using the variable selection strategy from Heinze and Dunkler.^28^ All statistically significant variables (i.e., P < 0.1) in the univariate analysis were included in the multivariable model to identify the independent contribution of each factor to dementia prevalence. The multivariable model was performed separately by sex and division. Results are reported as odds ratio (OR) with their 95% CI. In a sensitivity analysis, we re-ran the multivariable analysis incorporating age as a continuous (vs. categorical) variable in the model.^29^ A two-sided p-value of less than 0.05 indicated statistical significance. All analyses were carried out using IBM SPSS Statistics for Windows, Version 21.0. Armonk, NY: IBM Corp and R programming language.

### Ethics

The research proposal was approved by the Bangladesh Medical Research Council (BMRC) and Ethical Review Committee (ERC) of icddr,b. All study participants were recruited following written voluntary informed consent. Individuals identified with dementia were referred to the Department of Neurology at the tertiary care government teaching hospital located in each divisional city and received usual treatment free of cost.

### Role of the funding source

Not applicable.

## Results

### Population characteristics

Between January and December 2019, a total of 9219 households were listed across the seven administrative divisions and 4191 eligible participants were identified (45%). Of these eligible participants, 2800 participants were randomly selected for a revisit and 2795 were recruited (99% response rate), including 68% from rural areas. The total number of study participants was proportional to the population distribution in urban and rural areas in each division.^30^ As shown in Table 1, the mean age of the participants was 67 years ± 7 SD, 51% were female, 41% were single (divorce, separated or widowed), 42% had no education and 29% were employed. The distribution of education level, marital status and employment status significantly varied between male and female participants (Table 1).

**Table 1:** Socio-demographic characteristics of the older people (N = 2795).

	Overall		Male		Female		P-value (male vs. female)
	Total = 2795	(%)	n = 1369	48.9%	n = 1426	51.0%	
Age, Mean (SD)	67 ± 7		67.6 ± 7.2)		67.3 (±7.3)		0.431
Age group (In years)							
60–69 y	1882	67.3	910	66.5	972	68.2	0.657
70–79 y	695	24.9	347	25.3	348	24.4	
80–89 y	174	6.2	94	6.9	80	5.6	
90–115	44	1.6	18	1.3	26	1.8	
Education							
Never went to school	1171	41.9	480	35.1	691	48.5	P < 0.001
Some education	939	33.6	390	28.5	549	38.5	
Completed primary education	685	24.5	499	34.6	186	13.0	
Marital status							
Married	1642	58.7	1215	88.8	427	29.9	P < 0.001
Single^a^	1153	41.3	154	11.2	999	70.1	
Currently employed	813	29.1	680	49.7	133	9.3	P < 0.001
Socioeconomic status							
Poorest	448	16.0	231	16.9	217	15.2	0.566
Poor	522	18.7	246	18.0	276	19.4	
Middle	646	23.1	318	23.2	328	23.0	
Rich	566	20.3	283	20.7	283	19.8	
Richest	613	21.9	291	21.3	322	22.6	
Type of community							
Urban	896	32.0	450	32.9	446	31.3	0.360
Rural	1899	68.0	919	67.1	980	68.7	
Division							
Barishal	409	14.6	206	15.0	203	14.2	P < 0.001
Chattogram	341	12.2	144	10.5	197	14.0	
Dhaka	409	14.6	198	14.5	211	14.8	
Khulna	409	14.6	206	15.1	203	14.2	
Rajshahi	409	14.6	203	14.8	206	14.4	
Rangpur	409	14.7	239	17.5	170	11.9	
Sylhet	409	14.6	173	12.6	236	16.5	

a Single: Divorce/separated/widowed/unmarried.

Regional variations across seven divisions were observed in education, employment, marital status, and SES. Age distribution of the participants varied across the seven divisions, including more older aged people living in the Barisal division (mean age: 69 years ± 7 SD) and a younger cohort in the Rangpur division (mean age: 67 years ± 6 SD). The highest proportion of single was in Rajshahi (52.2%) and the lowest proportion was in Rangpur (28.4%). The Dhaka division had the highest proportion of participants who never went to school (50.6%) and the Khulna division had the lowest (27.4%). The highest proportion of participants employed in a job for living was in the Rajshahi division (47.2%) and the lowest proportion was in the Chattogram division (17.0%) (P < 0.001) (Appendix 3, Table S1).

### Prevalence of dementia

The mean MMSE score was 26.6 (SD: 2.5; Range: 9 to 30) and the overall estimated prevalence of dementia was 8.0% (95% CI: 7.0–8.9%; n = 223). The prevalence of dementia was positively correlated with age (r_pbc_: 0.164, P < 0.001) and negatively correlated with years of education (r_pbc_: −0.125, P < 0.001). A higher prevalence was observed among individuals aged ≥90 years compared to all other age groups combined (40.9% vs. 10.5%), among females than male (11.6% vs. 4.2%), among those who were single compared to those who were married (12.4% vs. 4.9%), among those with no education compared to those with any education (12.5% vs. 4.7%), among those who were unemployed compared to those employed (9.6% vs. 4.1%), and among those who belonged to a lower SES group compared to those who belonged to a higher SES group (9.4% vs. 8.6%). The overall prevalence of dementia significantly varied across the seven divisions, with the highest prevalence reported in Rajshahi (14.4%) and the lowest in Dhaka (2.9%) with no variation across urban and rural areas (Table 2). However, the prevalence of dementia was higher among females than males across age, education, occupation, marital status, and most of the divisions (except for Chattogram) (See Appendix 3, Figure S2).

**Table 2:** Prevalence of dementia across socio-demographic characteristics, type of community and divisions.

Variables	Total = 2795	With dementia (MMSE score <24)		Without dementia (MMSE score ≥24)		P-value^b^
		n = 223	%	n = 2573	%	
Gender						
Male	1369	57	4.2	1312	95.8	P < 0.001
Female	1426	166	11.6	1260	88.4	
Age group						
60–64 y	1183	59	5.0	1124	95.0	P < 0.001
65–69 y	699	42	6.0	657	94.0	
70–74 y	434	47	10.8	387	89.2	
75–79 y	261	31	11.9	230	88.1	
80–84 y	114	17	14.9	97	85.1	
85–89	60	9	15.0	51	85.0	
90–115	44	18	40.9	26	59.1	
Marital status						
Married	1642	80	4.9	1562	95.1	P < 0.001
Single^a^	1153	143	12.4	1010	87.6	
Education						
Completed priamry education	685	21	3.1	664	96.9	P < 0.001
Some education	939	56	6.0	883	94.0	
Never went to school	1171	146	12.5	1025	87.5	
Current engage in earning						
Yes	813	33	4.1	780	95.9	P < 0.001
No	1982	190	9.6	1792	90.4	
Place of residence						
Urban	896	70	7.8	826	92.2	0.824
Rural	1899	153	8.1	1746	91.9	
Socioeconomic status						
Lower	448	42	9.4	406	90.6	0.209
Lower middle	522	48	9.2	474	90.8	
Middle	646	41	6.3	605	93.7	
Upper middle	566	39	6.9	527	93.1	
Upper	613	53	8.6	560	91.4	
Division						
Rajshahi	409	59	14.4	350	85.6	P < 0.001
Rangpur	409	48	11.7	361	88.3	
Khulna	409	32	7.8	377	92.2	
Barisal	409	30	7.3	379	92.7	
Chattogram	341	23	6.7	318	93.3	
Sylhet	409	19	4.6	390	95.4	
Dhaka	409	12	2.9	397	97.1	

MMSE, Mini Mental State Examination.

a Widowed**,** Separated**,** Unmarried**,** Divorced.

b Applied chi-square test of independence.

### Projection of the dementia cases in 2025, 2041 and 2051

**Fig. 2:**
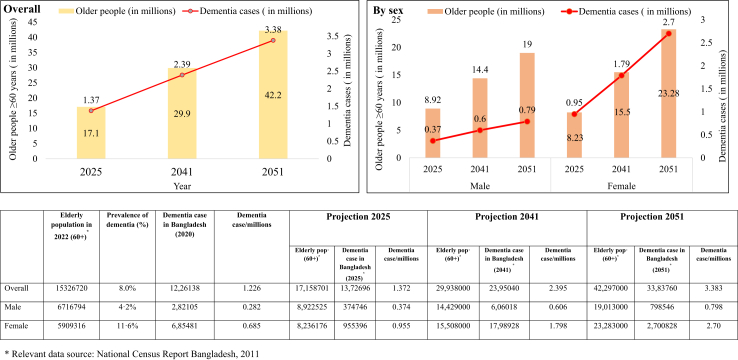
Projection of dementia case (2025–2050) among older age population (≥60 years) in Bangladesh.

The prevalence of dementia is estimated to be 1.2 million (800 cases per 10,000 population) in 2022 with females being at 2.8 times higher risk compared to males; including 0.7 million cases among females and 0.3 million cases among males. As shown in Fig. 2 it is estimated that the prevalence of dementia in Bangladesh will increase to 1.4 million cases in 2025 (including 0.4 million cases in males and 1.0 million cases in females), to 2.4 million cases in 2041 (including 0.6 million cases in males and 1.8 million cases in females), to 3.4 million cases in 2051 (including 0.8 million cases in males and 2.7 million cases in females) (Fig. 2).

### Bivariate analysis

The odds of dementia were higher in people aged ≥70 years (vs. people aged <70 years), in females (vs. males), in those who had no education (vs. who had any education), in single (vs. married), in those unemployed (vs. employed) and these differences were statistically significant (all P <0.1)*.* No association was observed between dementia and type of community (urban vs. rural) or SES (Appendix 3, Figure S2–S9).

### Multivariable analyses

**Fig. 3:**
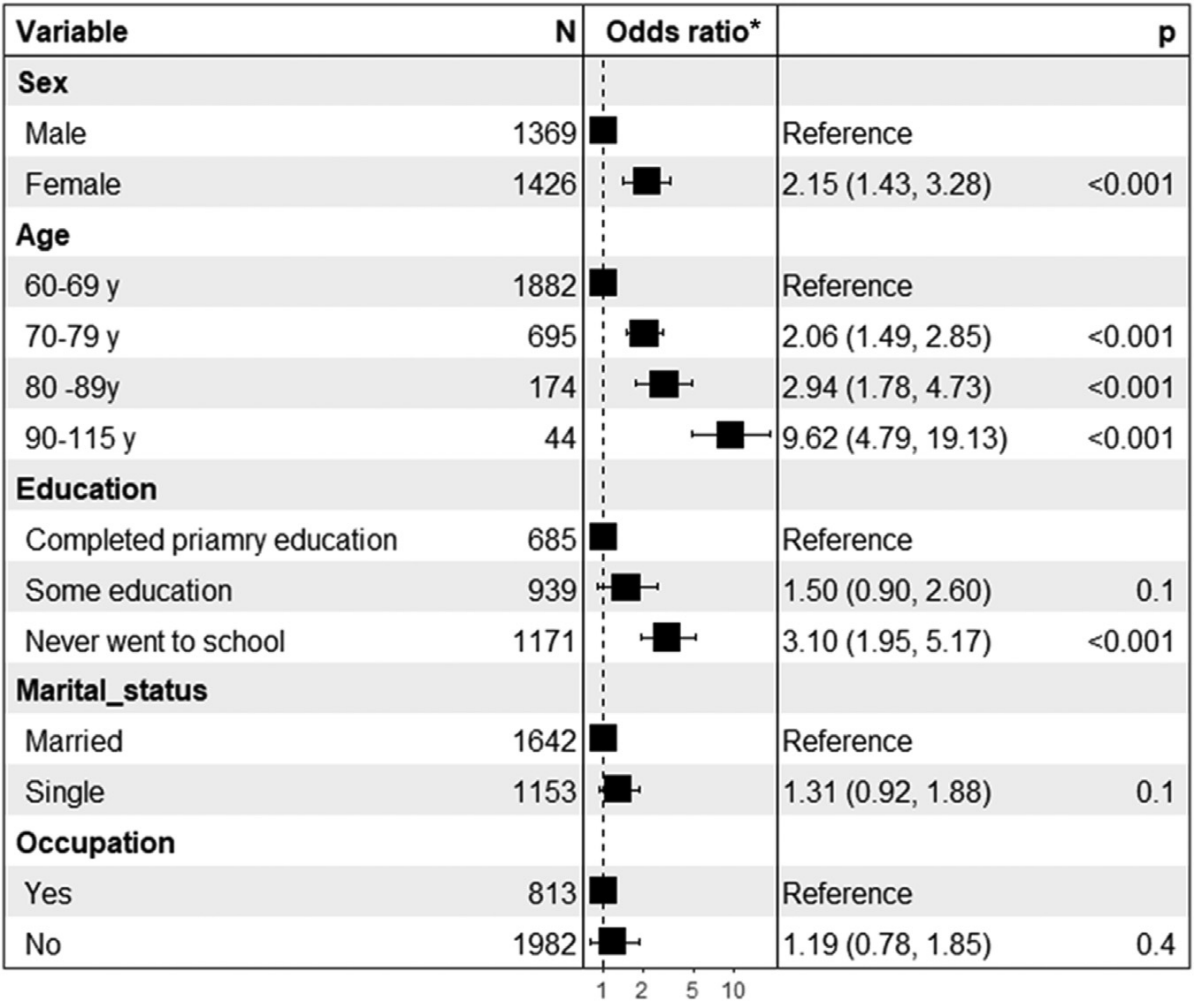
Factors associated with and without dementia of older people (∗Adjusted odds ratio with 95% CI.).

After adjusting for age, sex, education, marital status and occupation in a multivariable analysis, the odds of dementia were two times higher in females than males (OR: 2.2, 95% CI: 1.4–3.3), nine time higher among people aged ≥90 years compared to those aged 60–64 years (OR: 9.6, 95% CI: 4.8–19.1), and three times higher in people with no education compared to those who completed primary education (OR: 3.1, 95% CI: 20.0–5.2). Dementia prevalence was not associated with marital status (OR: 1.3, 95% CI: 0.9–1.9) or employment status (OR: 1.2, 95% CI: 0.8–1.9) in multivariable analysis. When age was entered into the model as a continuous variable, the odds of dementia increases by 6% per one-year increase in age, while the results for other factors remain unchanged (Fig. 3) (See Appendix 3, Figure S12).

In an interaction analysis, the odds of dementia were found to be eight times higher in females who never went to school (OR: 8.75, 95% CI: 4.19–18.24) when other variables were included in the model. However, the odds of dementia were found to be three times higher in males who never went to school (OR: 3.62, 95% CI: 1.69–7.75) when other variables were included in the model (See Appendix 3, Table S3).

### Sex stratified results

In bivariate analysis, the odds of dementia were three times higher in females than males (OR: 3.03, 95% CI: 2.24–4.17). When controlling for age, education, marital status, and occupation in the model, ‘increased age’ and ‘never went to school’ were significant risk factors for dementia in both of male and females. However, association between factors ‘being currently employed’ and marital status did not show any statistical significance (for male and female separately) (See Appendix 3, Figure S10).

### Division stratified results

In a similar multivariable analysis across divisions, people aged ≥70 years had higher odds of dementia in Chattogram, Barisal, Sylhet and Rajshahi compared to those in the younger age groups. Females had a higher odds of dementia in Khulna and Rajshahi compared to males. Also, people with no education had a higher odds of dementia in Khulna and Rangpur compared to those who had completed primary education. Furthermore, people classified as single had a higher odds of dementia in Rangpur and Dhaka compared to those who were married. However, occupation was not associated with dementia in any division (See Appendix 3, Figure S11).

## Discussion

This is the first study to estimate the prevalence of dementia at a national level and identify its key risk factors in the older population in Bangladesh. As highlighted by the results, the number of dementia cases is high, estimated at nearly one out of twelve people aged ≥60 years. By 2051, the number of cases is expected to almost double to approximately 3.4 million people. This study also demonstrates that the prevalence of dementia varies by key sociodemographic characteristics including age, sex, education, and marital status, but not SES. Dementia prevalence also varies across divisions suggesting regional variations, but we did not observe differences in prevalence by urban vs. rural location within the divisions.

When compared to other countries, the prevalence of dementia reported in Bangladesh (8.0%) is like that reported across the world (range 5.5–11.3%).^2^ However, when compared to estimates specifically from LMICs the prevalence of dementia is higher in Bangladesh than that reported in sub-Saharan Africa (5.5%) and other countries in Asia, including India, Pakistan and Cambodia (7.6%).^31^ Discrepancies in estimates across countries is a major concern, which might be due to differences in survival length, the distribution of risk/protective factors and design/methodology (e.g., sampling and dementia diagnostic criteria) across studies.^32^^,^^33^ Given the lack of dementia data in LMICs, including Bangladesh, the baseline evidence generated in this study is important to enable us to track disease course (i.e., with longitudinal follow-up), assess trends and assist with future planning for dementia risk reduction and prevention. The results could possibly be extrapolated to other LMICs which currently lack data but have similar country profiles (i.e., GDP/cultural/ethnic).

Life expectancy in Bangladesh has steadily increased from 66 years in 2000 to 73 years in 2019 indicating an increase in the number of older people.^34^ The projected number of people living with dementia is expected to more than double by 2051, which is in line with the current global projections over the next 20 years.^35^ This poses an unprecedented challenge for the future health system of Bangladesh. Currently there is no national level strategy focused on dementia care and only a few health facilities provide treatment for dementia. These include the National Institute of Neurosciences & Hospital, located in Dhaka, and the Department of Neurology of twenty government teaching hospitals located in urban areas.^36^ While a few private hospitals include specialty services for dementia these are prohibitively expensive and out of the reach for the majority of the population.^37^ Current health systems in Bangladesh are therefore not ready to tackle the vast number of current and future dementia cases. Work led by the 10/66 Dementia Research Group identified poverty, low levels of education and ageing as barriers to accessing healthcare among older people in LMICs.^38^ Further, like other LMICs, the opportunities for synergistic collaboration among health care professionals and policy makers are limited in Bangladesh due to a lack of research evidence. As such, it is imperative for Bangladesh, in addition to other LMICs, to develop a national action plan for dementia management and essential workforce development to establish a comprehensive dementia care model across divisions and in both urban and rural areas.

Our study has demonstrated that dementia prevalence was higher in females compared to males and supports findings reported globally.^12^^,^^39^^,^^40^ A possible reason for increased dementia risk in females may be increased life expectancy and biological differences (e.g., sex-specific hormones).^41^, ^42^, ^43^ Lower educational attainment in older females and the highest prevalence observed among those who never went to school might have negatively impacted cognitive reserve leading to an increased risk of dementia.^43^ We have observed that females were more likely to be single, supporting recent findings of a link between marital status and dementia.^44^^,^^45^

High educational attainment has consistently been associated with a lower risk of cognitive impairment and dementia via increased cognitive reserve in both high and LMIC settings.^33^^,^^43^ Indeed, for every one year increase in education attainment, the risk of dementia decreases by 7%.^43^^,^^46^ As such there is an urgent need to strength policy focused on improving access to formal education for all of the population of Bangladesh.^47^^,^^48^

Geographical variation in dementia prevalence has been reported in HICs and LMICs with higher prevalence typically observed in rural compared to urban areas.^49^^,^^50^ However, this effect was not observed in Bangladesh, which is unique. Reasons for this could be due to the distribution of sociodemographic characteristics (sex, age, and marital status) that did not vary across urban and rural sites. We did however observe variations in dementia prevalence across divisions, with the highest rates in Rajshahi and lowest in Dhaka. Geographical differences could be due to economic, educational, and healthcare related factors as well as variations in the distribution of risk factors (including marital status as documented here) across distinct locations.^32^^,^^51^

In this study, variations in prevalence of dementia were observed across divisions, and even after controlling for other sociodemographic characteristics, we found that age, female sex, and lower education level differences in dementia prevalence persisted across divisions. The findings are also indicating that dementia prevalence can be influenced by sociodemographic characteristics, which we were able to investigate in our study due to a large sample size that included divisional representation as well as the key sociodemographic factors.

The study has numerous strengths including a large sample size with representation across all administrative divisions in Bangladesh. We also recruited equal numbers of males and females at each study site. This allowed for an accurate estimate of dementia prevalence in the whole Bangladesh population as well as estimates stratified by sex and administrative division/geographic region. Such data are rare in LMICs including Bangladesh. This information is essential for policymakers, local government, and service providers to allow them to make evidence-based decisions focused on future research and care; and policy on dementia risk, risk reduction and prevention, specifically in Bangladesh.

However, the study has some limitations. First, the sample size of oldest-old individuals (i.e., people aged ≥80 years) was small and therefore, the age-specific prevalence of dementia in these age groups may be underestimated. However, the age distribution was representative of the Bangladesh population, as highlighted above. Second, among the study population >40% of participants never went to school and some could not hold a pen. For some individuals, this prohibited the ability to complete the MMSE item requesting the drawing of a box, which was subsequently scored as 0. Therefore, it is possible that the classification of dementia for these individuals was incorrect. However, to avoid incorrect diagnosis, a study doctor examined all borderline cases (i.e., people with an MMSE = 24). Third, although the MMSE scale has been customised for Bangladesh,^52^ scoring does not take into account factors such as age, cultural norms, literacy level, and pre morbid cognitive ability all of which can influence an individual's MMSE score.^53^, ^54^, ^55^ Thus, the low literacy level of the participants along with a high unemployment rate, particularly among the females, might have influenced responses to certain questions and resulted in floor effects.^56^^,^^57^ Fourth, the MMSE is a screening test for global cognitive function and a comprehensive clinical assessment including a full neuropsychological evaluation is recommended for a dementia diagnosis. However, given the scale of this study such testing was not possible. Last, due to natural disasters including heavy rainfall during the study testing period, the sample size in the Chattogram division was 15% lower than the target of n = 400. However, the total number of recruited participants was higher than the lowest estimated sample size of 340, which was calculated to be sufficient for estimating dementia prevalence in each division.

There is an urgent need for large scale, population-representative research focused on ageing and dementia in LMICs, including Bangladesh. Future studies should include a validated MMSE test in addition to more in-depth interviewing, using for example the 10/66 interview schedule,^5^ to enhance dementia diagnosis and allow for cross-study comparability. In addition, a minimum set of variables needs to be agreed for all studies (e.g., focused on sociodemographic, health, lifestyle and LMIC context-specific factors such as food security, diet, environmental and social context). This will allow for comparison across sites of key risk and protective factors essential for informing the development of context and culturally specific intervention strategies. Furthermore, funding needs to be made available to support longitudinal follow-up of study participants to inform analysis focused on disease trajectories and informing regional variations over time.

In Bangladesh, the prevalence of dementia is high particularly in older people, females, and those with no education. There is therefore an urgent need to focus resources and policy on prevention, risk reduction and the improvement of care for people with dementia to mitigate the social, personal, and economic impacts of this condition. However, in low-resource settings, such as Bangladesh, this is difficult; particularly when there is little research into dementia prevalence and risk. The results here provide the first step towards informing an evidence-based national dementia action plan that is unique to the context of Bangladesh including social and cultural norms as well as resource availability.

## Contributors

AN developed the concept, designed methodology, obtained funding, supervised project implementation, conducted data analysis, and wrote the manuscript. MSI conducted formal data analysis and reviewed literature. MSI and AN developed the first draft. MH, MBI, AAP, MRA, BCS, QDM, MRA, BCS and QDM contributed to developing methodology and interpretations of findings. EYHT contributed to interpretation of findings and manuscript editing. All authors contributed to the manuscript and approved the final manuscript.

## Data sharing statement

The data extracted from this study can be obtained from the corresponding author upon reasonable request.

## Editor note

The Lancet Group takes a neutral position with respect to territorial claims in published maps and institutional affiliations.

## Declaration of interests

None.
