# Supplementary material for: Prevalence of dementia among older age people and variation across different sociodemographic characteristics: a cross-sectional study in Bangladesh
Source: Lancet Reg Health Southeast Asia. 2023 Aug 24;17:100257. doi: 10.1016/j.lansea.2023.100257 (PMC10577143; doi:10.1016/j.lansea.2023.100257)
Supplement: Appendix 1 [file mmc1.docx]

**Appendix 1**

**Figure S1:** Recruitment of the study participants (flow diagram)


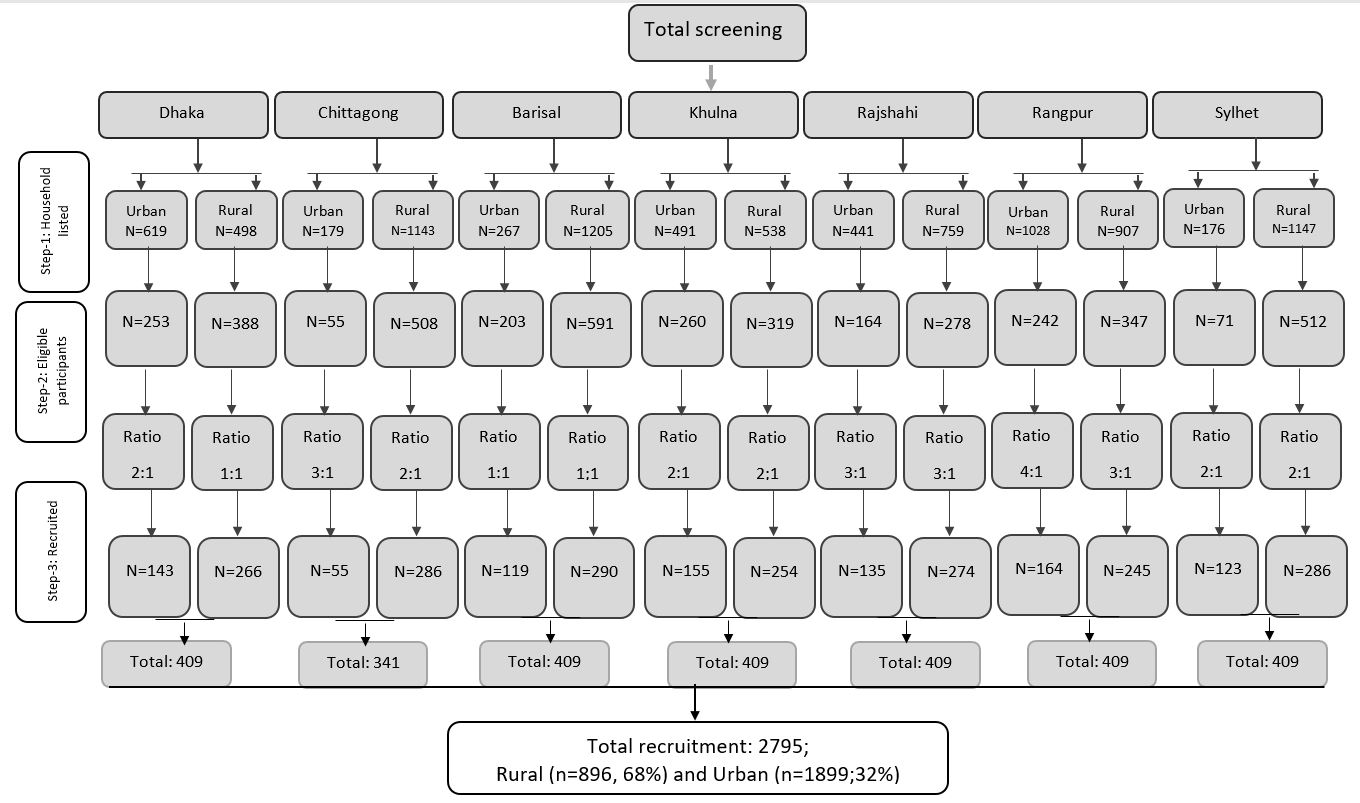


1. Eligible participants: 60 years and above;
2. Ratio: Household listed/ eligible participants
3. Average number of households visited to get one eligible participant: Three households in an urban area (range: 01-04) and two households in a rural area (range: 01-03).
