# Supplementary material for: Prevalence of dementia among older age people and variation across different sociodemographic characteristics: a cross-sectional study in Bangladesh
Source: Lancet Reg Health Southeast Asia. 2023 Aug 24;17:100257. doi: 10.1016/j.lansea.2023.100257 (PMC10577143; doi:10.1016/j.lansea.2023.100257)
Supplement: Appendix 2 [file mmc2.docx]

**Appendix 2**

*Methodological procedures of calculating wealth index*

The wealth index (WI) calculation procedure was developed by the Demography and Health Survey (DHS) program and it is globally accepted for use in low-income and middle-income countries. The following steps have been applied for calculating wealth index in this study.

Step 1. The household asset variables included the source of drinking water, type of toilet facilities, type of floor, wall, and roof, which were converted into indicator variables (dichotomous) by coding 0 or 1. An indicator variable was coded as 1 if the item was available in the household and 0 otherwise. Number of persons per sleeping room was considered as a continuous variable. We ran simple frequency of the original variables to compare with the newly created indicator variables and corrected any inconsistencies observed.

Step 2. A set of common indicator variables was selected based on published evidence derived from the published reports of Bangladesh Demography and Health Survey (BDHS). Principal Components Analysis (PCA) was applied on the common indicator variables to create a common wealth score for a household following correlation method. To construct a wealth score for an urban household, we applied PCA on the indicator variables of the urban households and applied the same technique on the indicator variables of the rural households for creating a wealth score for a rural household.

Step 3. Regression analysis was performed using common wealth score as an independent variable and urban wealth scores as dependent variable in the model, and generated a composite wealth score for the urban households. The composite wealth score of each household was ranked based on the number of household members to derive a wealth index (WI) for each of the urban households. The ranking of WI was then divided into five equal quintiles (20%) to label an urban household under one of the five socio economic groups; lower, lower middle, middle, upper middle, and upper. Similar processes were followed for generating a composite wealth score for the rural households using common wealth score and rural wealth scores for ranking the rural households under one of the five socio economic groups.
