# Supplementary material for: Prevalence of dementia among older age people and variation across different sociodemographic characteristics: a cross-sectional study in Bangladesh
Source: Lancet Reg Health Southeast Asia. 2023 Aug 24;17:100257. doi: 10.1016/j.lansea.2023.100257 (PMC10577143; doi:10.1016/j.lansea.2023.100257)
Supplement: Appendix 3 [file mmc3.docx]

**Appendix 3**

**Table S1.** Bivariate analysis of dementia with socio-demographic characteristics and divisions

|  | Dhaka | | Chattogram | | Barisal | | Khulna | | Rajshahi | | Rangpur | | Sylhet | | P-value |
| --- | --- | --- | --- | --- | --- | --- | --- | --- | --- | --- | --- | --- | --- | --- | --- |
|  | N=409 | % | N=  341 | % | N=  409 | % | N=  409 | % | N=  409 | % | N=409 | % | N=  409 | % |  |
| Age, median (range) | 66 (60-100) | | 65 (60-115) | | 66 (60-105) | | 66 (60-95) | | 65 (60-98) | | 65 (60-99) | | 65 (60-99) | | 0·067^[[1]](#footnote-1)^ |
| Age group |  |  |  |  |  |  |  |  |  |  |  |  |  |  |  |
| 60-69 years | 293 | 71.6 | 221 | 64.8 | 253 | 61.9 | 289 | 70.7 | 266 | 65.0 | 283 | 69.2 | 277 | 67.7 | Ref. |
| 70-79 years | 90 | 22.0 | 87 | 25.5 | 114 | 27.9 | 94 | 23.0 | 99 | 24.2 | 108 | 26.4 | 103 | 25.2 | 0.325 |
| 80 -89years | 21 | 5.1 | 24 | 7.0 | 35 | 8.6 | 23 | 5.6 | 38 | 9.3 | 12 | 2.9 | 21 | 5.1 | 0.001 |
| 90-115 years | 5 | 1.2 | 9 | 2.6 | 7 | 1.7 | 3 | .7 | 6 | 1.5 | 6 | 1.5 | 8 | 2.0 | 0.435 |
| Marital status |  |  |  |  |  |  |  |  |  |  |  |  |  |  |  |
| Single | 154 | 37·7 | 178 | 52·2 | 164 | 40·1 | 170 | 41·6 | 172 | 42·1 | 116 | 28·4 | 199 | 48·7 | P<0·001 |
| Married | 255 | 62·3 | 163 | 47·8 | 245 | 59·9 | 239 | 58·4 | 237 | 57·9 | 293 | 71·6 | 210 | 51·3 |  |
| Education level |  |  |  |  |  |  |  |  |  |  |  |  |  |  |  |
| Never went to school | 207 | 50·6 | 141 | 41·3 | 112 | 27·4 | 108 | 26·4 | 201 | 49·1 | 202 | 49·4 | 200 | 48·9 | Ref· |
| Some education (1 to 4 grade) | 134 | 32·8 | 126 | 37·0 | 138 | 33·7 | 155 | 37·9 | 125 | 30·6 | 133 | 32·5 | 128 | 31·3 | P<0·001 |
| Completed primary education | 68 | 16·6 | 74 | 21·7 | 159 | 38·9 | 146 | 35·7 | 83 | 20·3 | 74 | 18·1 | 81 | 19·8 | P<0·001 |
| Currently employed | 114 | 27·9 | 58 | 17·0 | 118 | 28·9 | 124 | 30·3 | 131 | 32·0 | 193 | 47·2 | 75 | 18·3 | P<0·001 |
| HH earning member, mean (SD) | 2 (0·8) |  | 2 (1·1) |  | 1 (0·9) |  | 1 (0·9) |  | 1 (0·8) |  | 1 (0·8) |  | 2 (1) |  | 0.892 |
| Socioeconomic status |  |  |  |  |  |  |  |  |  |  |  |  |  |  |  |
| Lower | 56 | 13·7 | 63 | 18·5 | 47 | 11·5 | 65 | 15·9 | 80 | 19·6 | 63 | 15·4 | 74 | 18·1 | Ref· |
| Lower middle | 33 | 8·1 | 37 | 10·9 | 107 | 26·2 | 98 | 24·0 | 82 | 20·0 | 99 | 24·2 | 66 | 16·1 | P<0·001 |
| Middle | 152 | 37·2 | 81 | 23·8 | 87 | 21·3 | 81 | 19·8 | 71 | 17·4 | 74 | 18·1 | 100 | 24·4 | P<0·001 |
| Upper middle | 86 | 21·0 | 92 | 27·0 | 85 | 20·8 | 64 | 15·6 | 86 | 21·0 | 66 | 16·1 | 87 | 21·3 | 0·107 |
| ^1^The Kruskal–Wallis one-way ANOVA. HH = household. | | | | | | | | | | | | | | | |

**Figure S2.** Prevalence of dementia across socio-demographic characteristics; (a) the x-axis represents different socio-demographic group; (b) the y-axis represents the proportion of dementia

| (a) *Dementia prevalence by Age* | *(b) Dementia prevalence by education level* |
| --- | --- |
| P<0·001  P=0·007  P=0·004  P<0·001 |  |
| c) Dementia prevalence by marital status | (d) Dementia prevalence by occupation |
|  | P<0.001  P<0.001 |
| (e) Dementia prevalence by type of community | (e) Dementia prevalence by socio-economic status |
|  |  |
| ( f ) Dementai prevalence by division |  |
| P=0.026  P=0.017  P<0.001  P=0.001  P=0.775  P=0.007  P<0.001 | |

***Bivariate analysis between socio demographic characteristics with the prevalence of dementia***

**Table S2.** Bivariate analyses between socio-demographic characteristics with and without dementia of older age populations

| Variables | With Dementia | | Without dementia | | Unadjusted OR (95 % CI) |
| --- | --- | --- | --- | --- | --- |
|  | n=223 | % | n=2573 | % |  |
| Sex |  |  |  |  |  |
| Male | 57 | 25.6 | 1312 | 51.0 | Ref. |
| Female | 166 | 74.4 | 1261 | 49.0 | 3.03(2.22-4.13)^*^ |
| Age group |  |  |  |  |  |
| 60-69 y | 101 | 45.3 | 1781 | 69.2 | Ref. |
| 70-79 y | 78 | 35.0 | 617 | 24.0 | 2.23(1.64-3.04) |
| 80-89 y | 44 | 19.7 | 174 | 6.8 | 3.09(1.95-4.91) |
| ≥90 y |  |  |  |  | 12.02 (6.47-23.0) |
| Marital status |  |  |  |  |  |
| Married | 143 | 64.1 | 1011 | 39.3 | Ref. |
| Single^#^ | 80 | 35.9 | 1562 | 60.7 | 2.76(2.08-3.67)^*^ |
| Education |  |  |  |  |  |
| Completed primary education | 21 | 9.4 | 664 | 25.8 | 2.01(1.2-3.34)^*^ |
| Some education | 56 | 25.1 | 883 | 34.3 | 4.5(2.82-7.19)^*^ |
| Never went to school | 146 | 65.5 | 1025 | 39.9 | Ref. |
| Marital status |  |  |  |  |  |
| Married | 80 | 35.9 | 1562 | 60.7 | Ref. |
| Single | 143 | 64.1 | 1010 | 39.3 | 2.76(2.08-3.68)^*^ |
| Occupation |  |  |  |  |  |
| Employed | 33 | 14.8 | 780 | 30.3 | Ref. |
| Unemployed | 190 | 85.2 | 1792 | 69.7 | 2.51(1.72-3.67)^*^ |
| Place of residence |  |  |  |  |  |
| Urban | 70 | 31.4 | 826 | 32.1 | Ref. |
| Rural | 153 | 68.6 | 1746 | 67.9 | 1.03 (0.77-1.38) |
| Socioeconomic status |  |  |  |  |  |
| Lower | 42 | 18.8 | 406 | 15.8 | 1.09(0.71-1.67) |
| Lower middle | 48 | 21.5 | 474 | 18.4 | 1.07(0.71-1.61) |
| Middle | 41 | 18.4 | 605 | 23.5 | 0.72(0.47-1.09) |
| Upper middle | 39 | 17.5 | 527 | 20.5 | 0.78(0.51-1.2) |
| Upper | 53 | 23.8 | 560 | 21.8 | Ref. |
| Division |  |  |  |  |  |
| Dhaka | 12 | 5.4 | 397 | 15.4 | Ref. |
| Chattogram | 23 | 10.3 | 318 | 12.4 | 2.39(1.17-4.88) |
| Barisal | 30 | 13.5 | 379 | 14.7 | 2.62(1.32-5.19) |
| Khulna | 32 | 14.3 | 377 | 14.7 | 2.81(1.43-5.53) |
| Rajshahi | 59 | 26.5 | 350 | 13.6 | 5.58(2.95-10.55) |
| Rangpur | 48 | 21.5 | 361 | 14.0 | 4.4(2.3-8.41) |
| Sylhet | 19 | 8.5 | 390 | 15.2 | 1.61(0.77-3.36) |

**^#^** Widowed**,** Separated**,** Unmarried**,** Divorced; ^*^ Statistically significant at P<0.1

***Sensitivity analysis for the selection of variables in multivariable analysis***

**Figure S3.** Association between dementia and age adjusted with different socio-demographic characteristics.

Age: The odds of dementia were two times higher in people aged 70 to 79 years, three times higher in people aged 80 to 89 years and twelves times higher in people aged 90 years and older compared to people aged 60 to 64 years. The pattern remained unchanged for people across all age groups when sex, education, SES and type of community were individually adjusted. However, this pattern of association was slightly lower, but still highly significantly associated when adjusted for marital status and occupation (Appendix Figure S3).

| **(a)Dementia~ Age groups** | **(b)Dementia~ Age groups+ Sex** | **(c )Dementia~ Age groups+ Education** |
| --- | --- | --- |
| 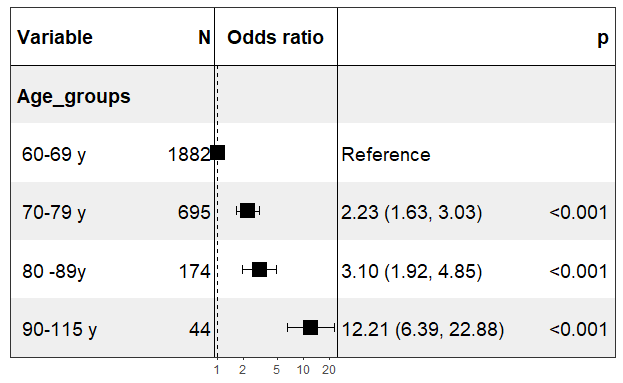 | 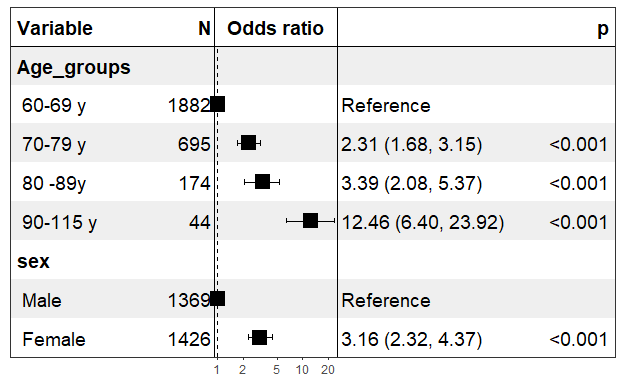 | 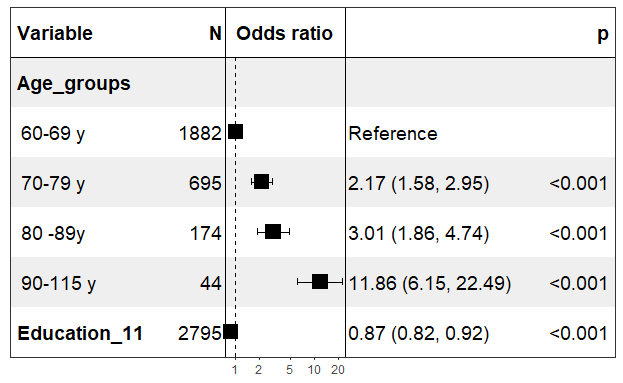 |
| **(d)Dementia~ Age groups+ Marita status** | **(e) Dementia~ Age groups+ Occupation** | **(f) Dementia~ Age groups+ Type of community** |
| 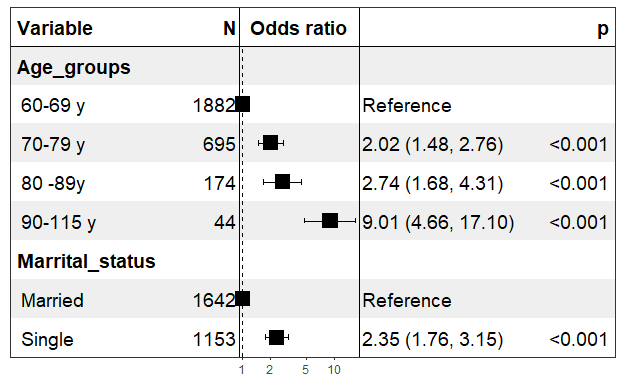 | 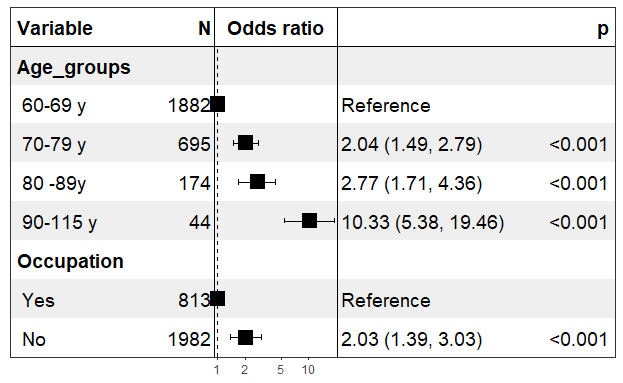 | 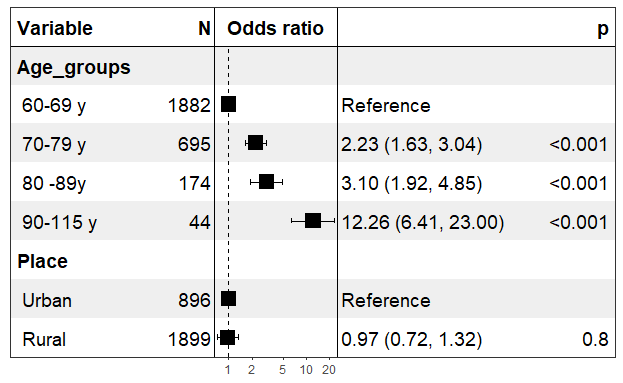 |
| (g) **Dementia~age+Socioeconomic status** |  |  |
| 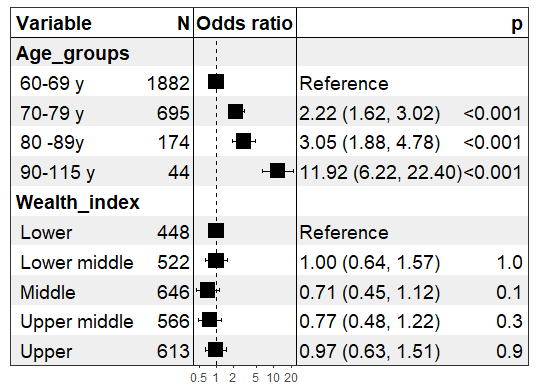 |  |  |

*Sex*

The odds of dementia were three times higher in females than males (OR:3·03, 95% CI:2·24-4·17). The pattern remained unchanged when age, SES and type of community were individually adjusted for, but two times higher when education, marital status and occupation were adjusted in the model (Appendix figure S3).

**Figure S4.** Association between dementia and sex adjusted with different socio-demographic characteristics

| **(a) Dementia~ Sex** | **(b) Dementia~ Sex +Age** | **© Dementia~ Sex +Education in years)** |
| --- | --- | --- |
| 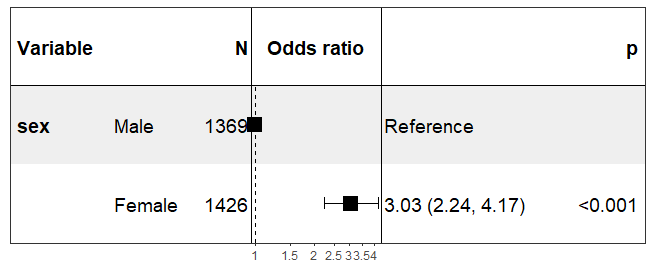 | 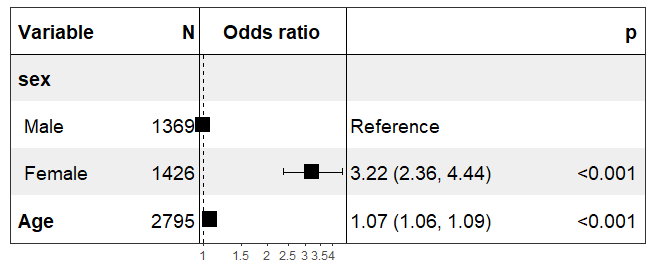 | 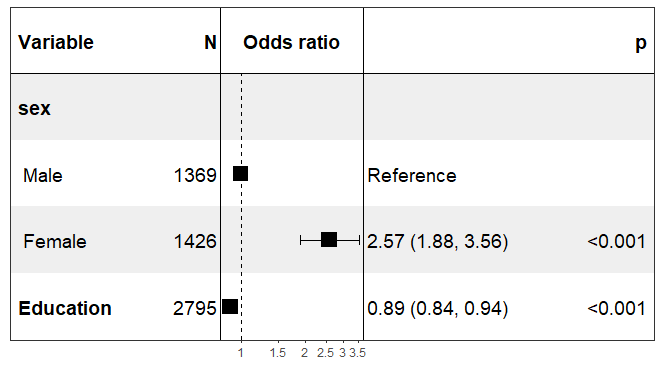 |
| **(d) Dementia~ Sex + Marital status** | **(e) Dementia~ Sex +Occupation** | **(f) Dementia~ Sex +** Type of community |
| 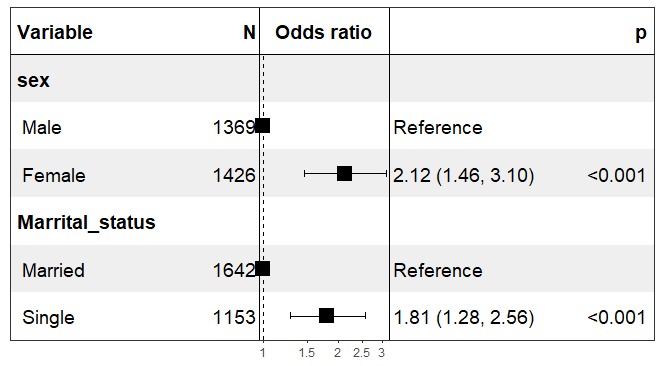 | 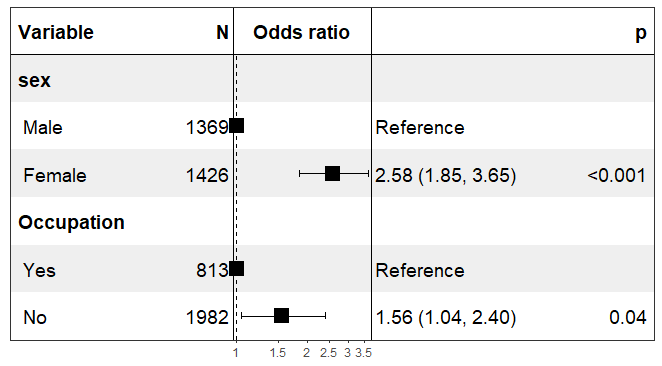 | 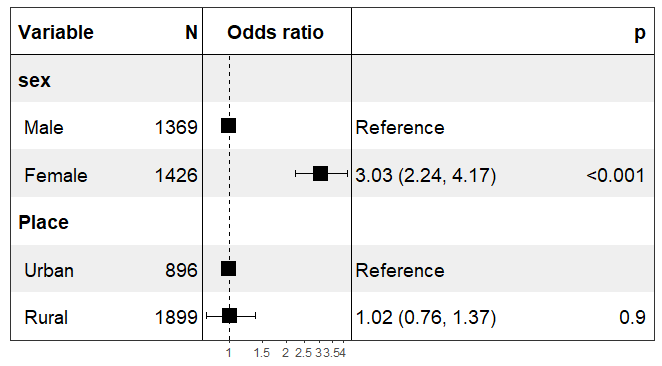 |
| **(g) Dementia~Sex+socioeconomic status** |  |  |
| 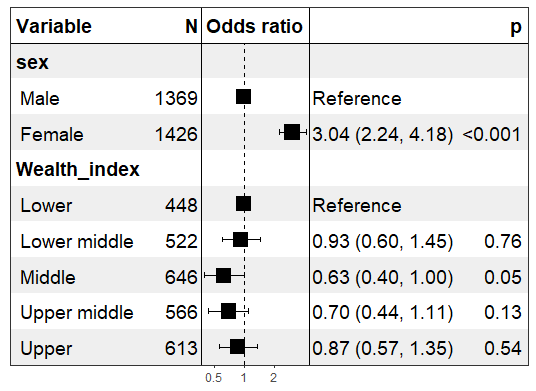 |  |  |

*Education*

The odds of dementia were four times higher in people with no education (OR:4·50 ,95% CI:2·89-7·39) and two times higher in those who had some education (OR: 2·01, 95% CI:1·22-3·42) compared to those who had completed primary education. This pattern of association remains unchanged when age, SES, occupation and type of community were individually adjusted for. However, the odds of dementia were three times higher for no education after adjusting for sex and marital status, but less than two times higher for some education compared to those completed primary education (appendix figure S4).

**Figure S5.** Association between dementia and education adjusted with different socio-demographic characteristics

| **(a) Dementia~ Education** | **(b) Dementia~ Education +Sex** | **(c ) Dementia~ Education +Age** |
| --- | --- | --- |
| 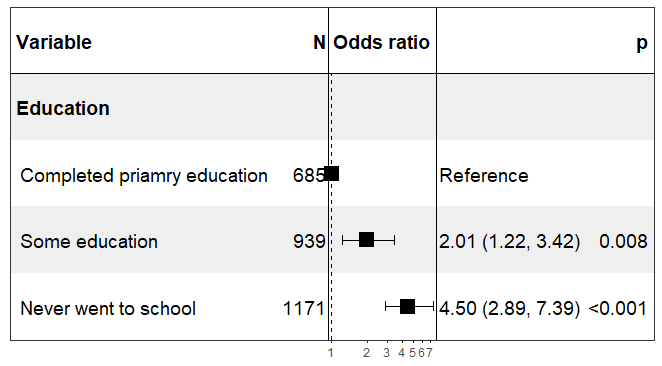 | 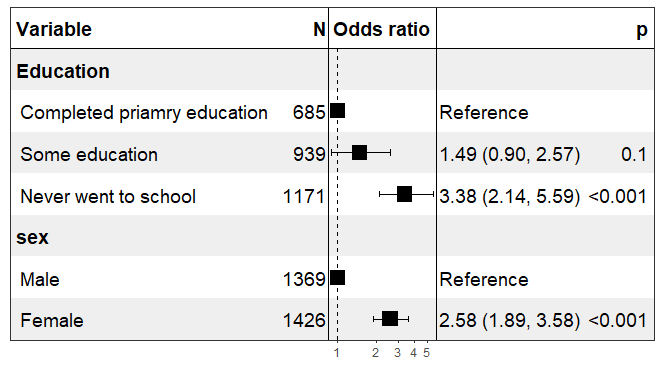 | 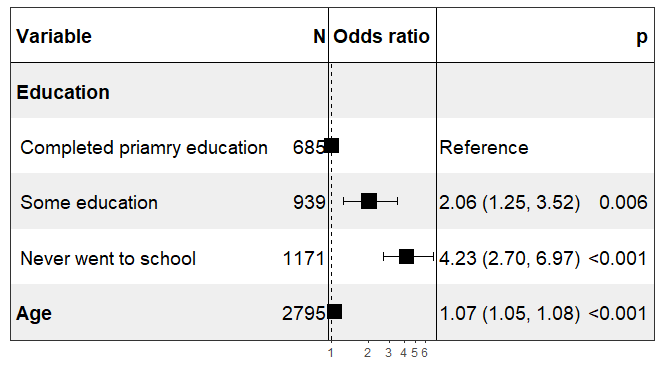 |
| **(d)Dementia~ Education+ marital status** | **(e) Dementia~ Education+ Occupation** | **(f) Dementia~ Education+ Type of community** |
| 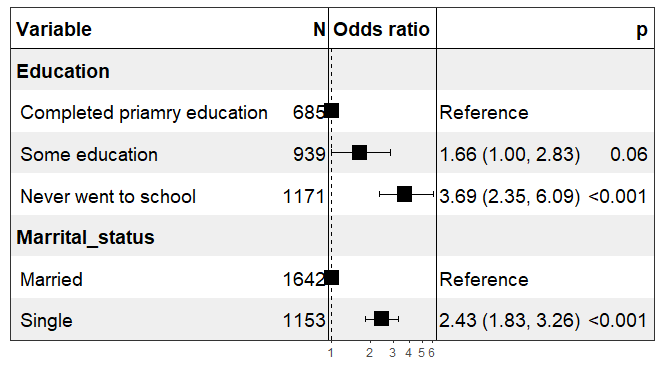 | 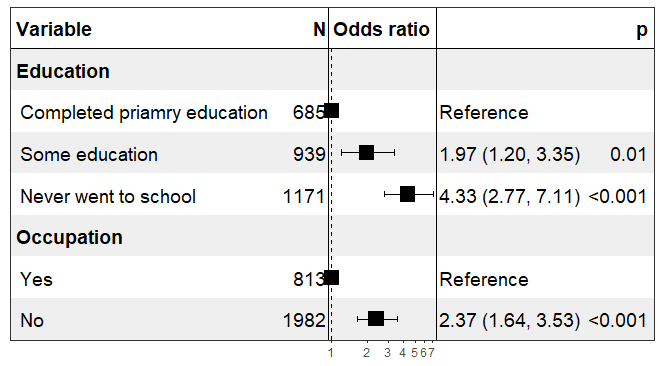 | 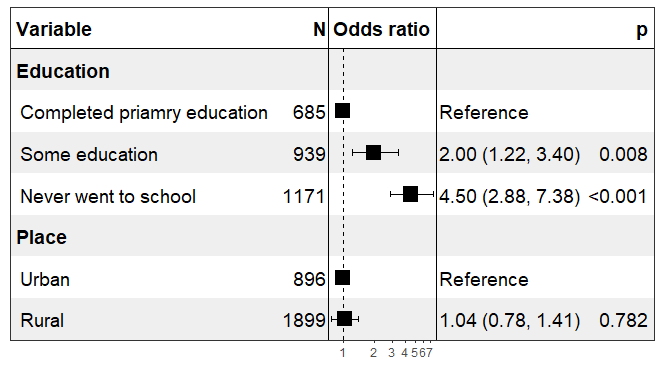 |
| **(g) Dementia~Education+Socioeconomic Status** |  |  |
| 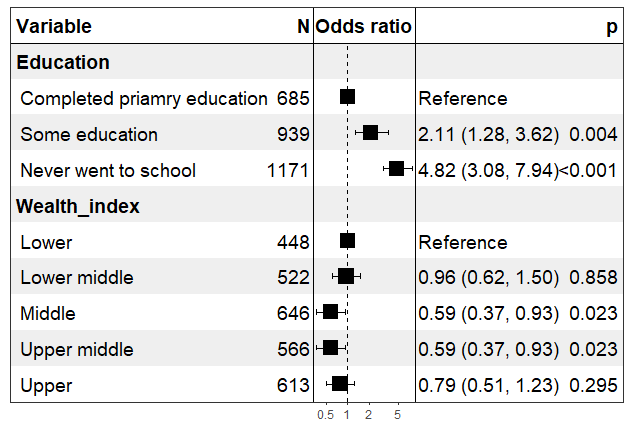 |  |  |

*Employment*

The odds of dementia were two times higher in those who were not employed compared to those who were employed at the time of the survey (OR: 2·51, 95% CI:1·74-3·72). This pattern of association remained unchanged when age, education, SES, and type community were individually adjusted for. However, the odds of dementia were less than two times higher when sex and marital status were individually adjusted for (Appendix figure S5).

**Figure S6.** Association between dementia and occupations adjusted with different socio-demographic characteristics

| **(a) Dementia~ Occupation** | **(b) Dementia~ Occupation+ Sex** | **(c) Dementia~ Occupation+ Age** |
| --- | --- | --- |
| 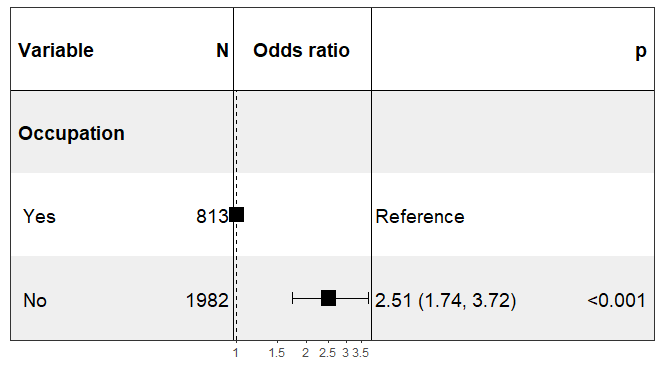 | 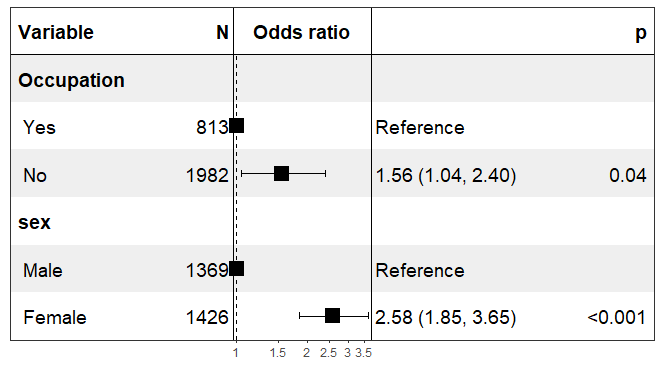 | 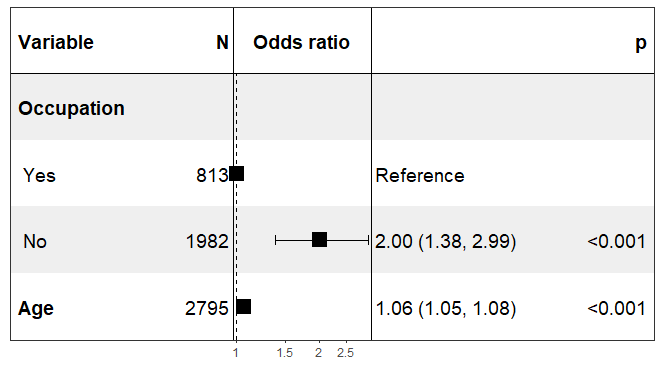 |
| **(d) Dementia~ Occupation+ Education** | **(e) Dementia~ Occupation+ Marital status** | **(f) Dementia~ Occupation+** Type of community |
| 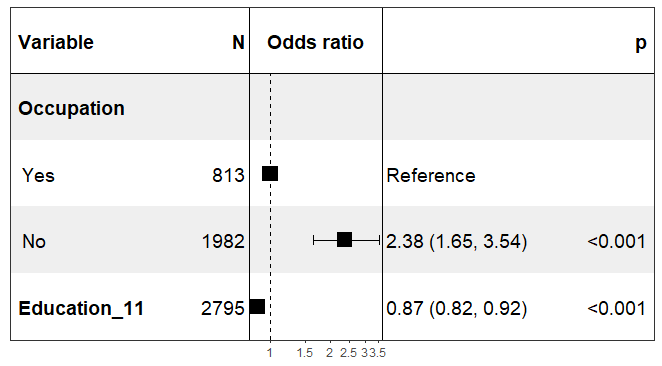 | 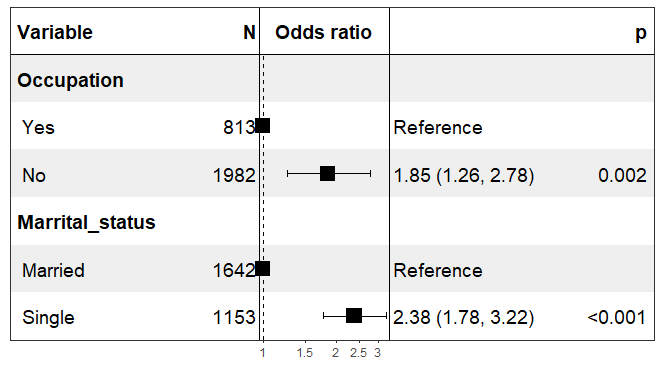 | 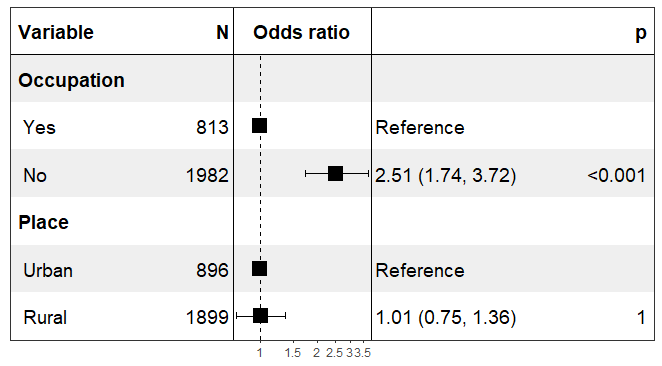 |
| **(g) Dementai~Occupation+Socioeconomic status** |  |  |
| 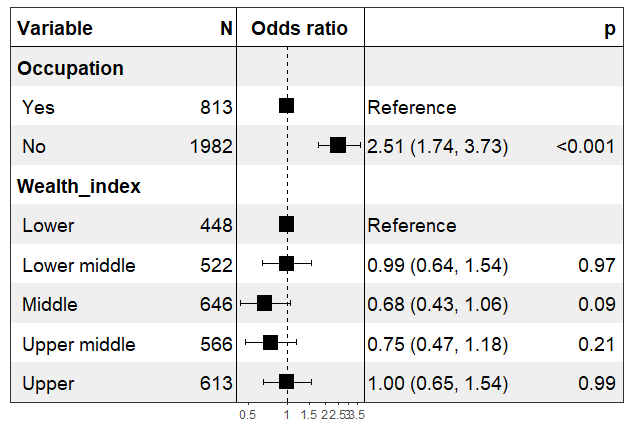 |  |  |

*Marital status*

In bivariate analysis, the odds of dementia were two times higher among single compared to married people (OR:2·76, 95% CI:2·09-3·69). This relationship sustained when age, education, SES, occupation, and type of community were individually adjusted for, but was observed to be lower after adjusting for sex (Appendix Figure S6)**.**

**Figure S7.** Association between dementia and marital status adjusted with different socio-demographic characteristics

| **(a) Dementia~ Marital status** | **(b)Dementia~ Marital status+ Age** | **© Dementia~ Marital status+ Sex** |
| --- | --- | --- |
| 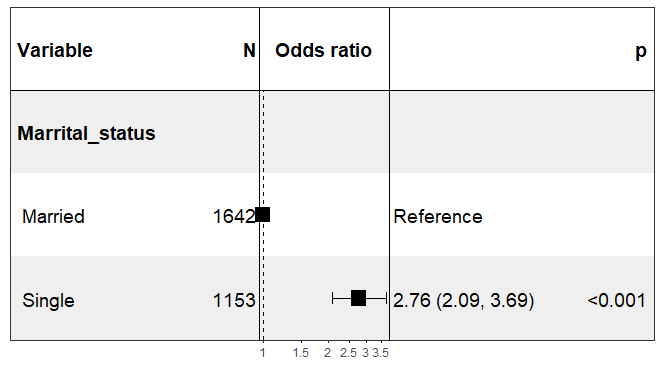 | 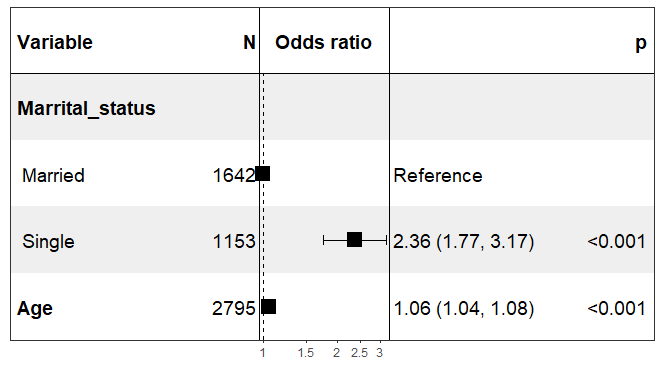 | 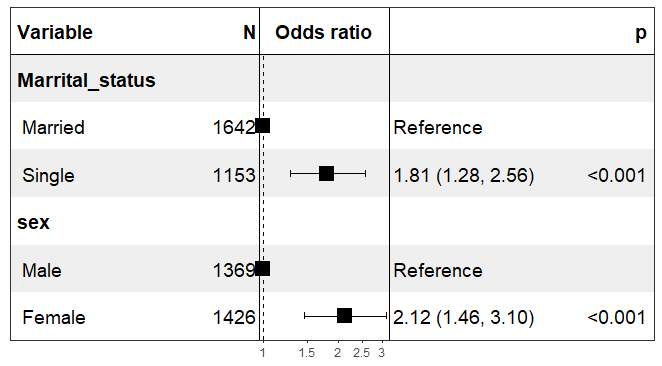 |
| **(d) Dementia~ Marital status+ Education** | **(e) Dementia~ Marital status+ Occupation** | **(f) Dementia~ Marital status+** Type of community |
| 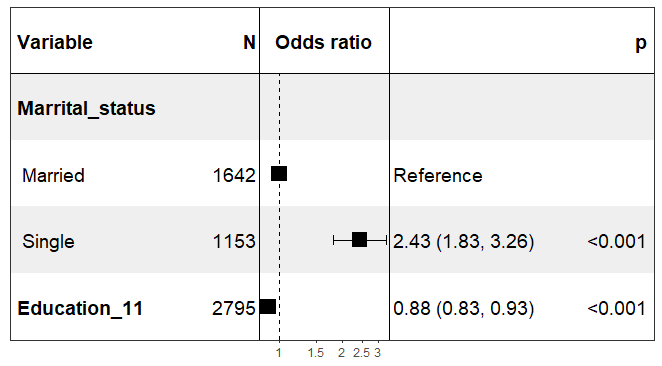 | 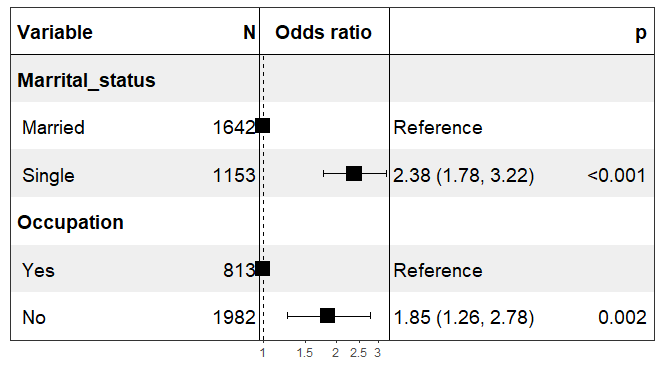 | 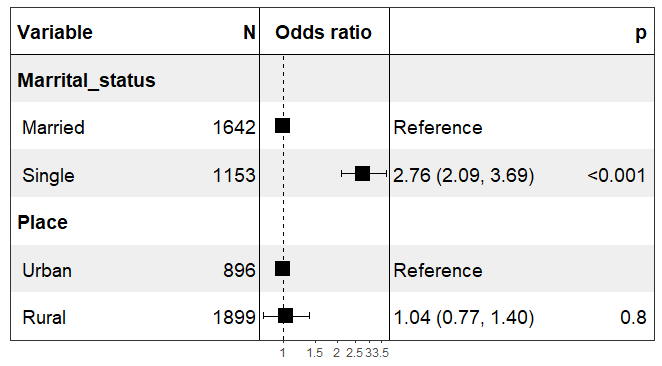 |
| **(g) Dementia~ Marital status+ Socioeconomic status** |  |  |
| 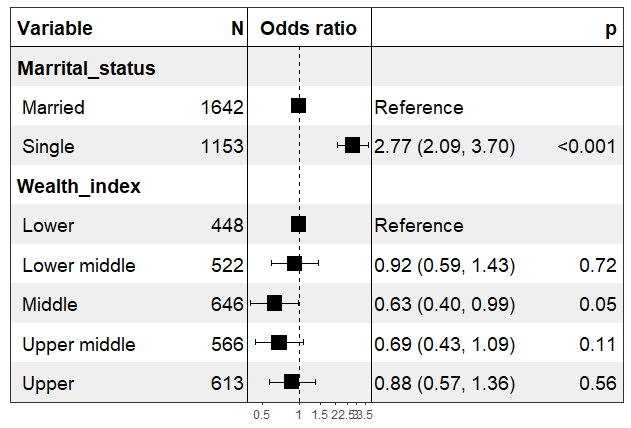 |  |  |

*Type of community*

There was no significant association between the type of community (urban vs· rural) (OR:1·03, 95% CI:0·77-1·38) in bivariate analysis, which remain unchanged when others socio-demographic factors were adjusted separately (Appendix Figure S7)**.**

**Figure S8.** Association between dementia and marital status adjusted with different socio-demographic characteristics

| **(a) Dementia~ Type of community** | **(b) Dementia~ Type of community+ Age** | **© Dementia~ Type of community + Sex** |
| --- | --- | --- |
| 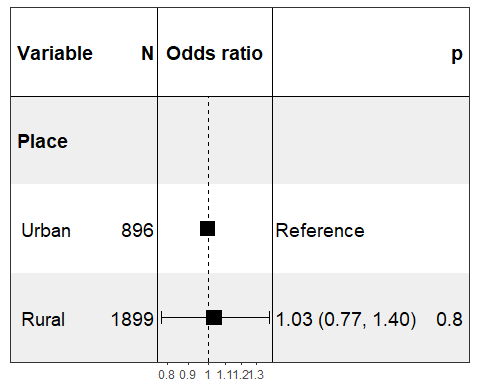 | 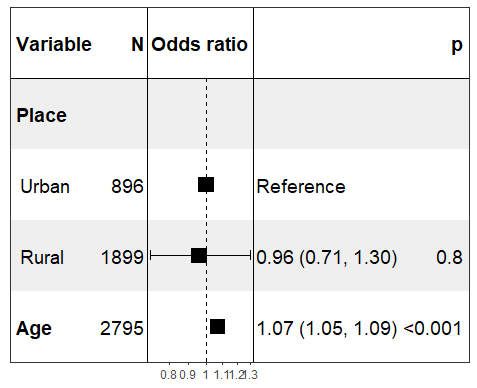 | 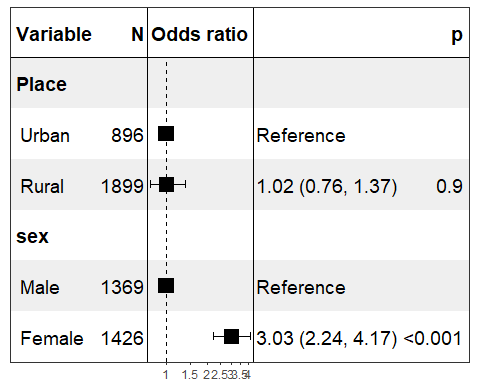 |
| **(d) Dementia~ Type community + Education** | **(e) Dementia~ Type of community + Occupation** | **(f) Dementia~ Type of community +** Marital status |
| 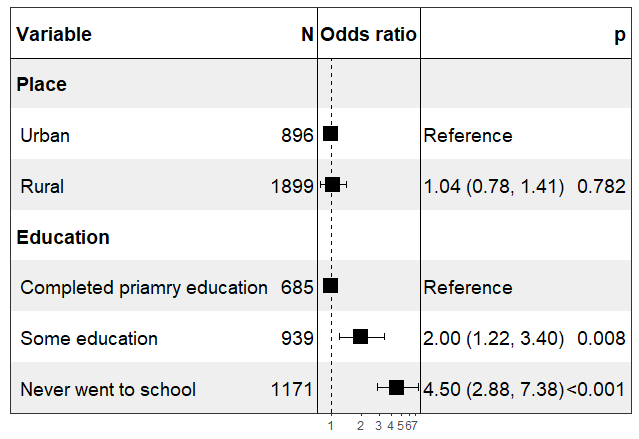 | 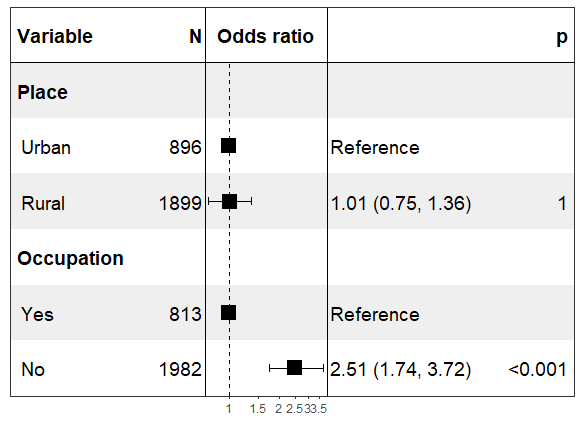 | 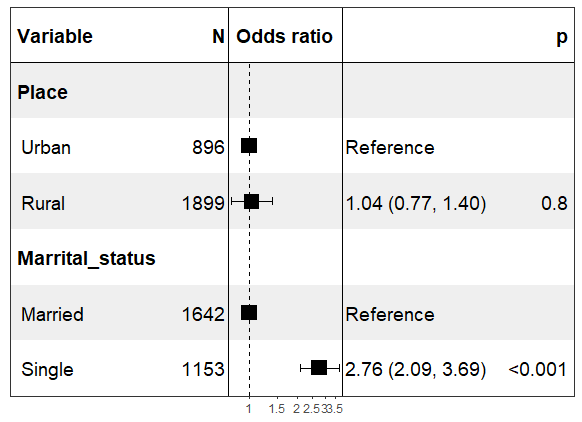 |
| **(g) Dementia~ Type of community + Socioeconomic status** |  |  |
| 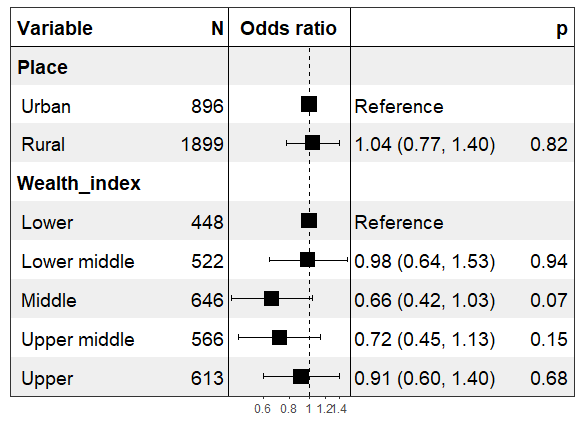 |  |  |

*Socio-economic status (SES)*

Overall, there was no association between dementia and socioeconomic status, and this relationship remained unchanged when others socio-demographic factors were individually adjusted, except for education (Appendix Figure S8).

**Figure S9.** Association between dementia and marital status adjusted with different socio-demographic characteristics

| **(a) Dementia~ Socioeconomic status** | **(b) Dementia~ Socioeconomic status + Age** | **(c) Dementia~ Socioeconomic status + Sex** |
| --- | --- | --- |
| 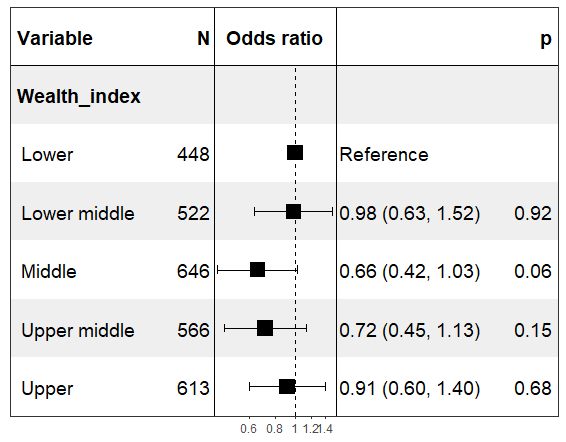 | 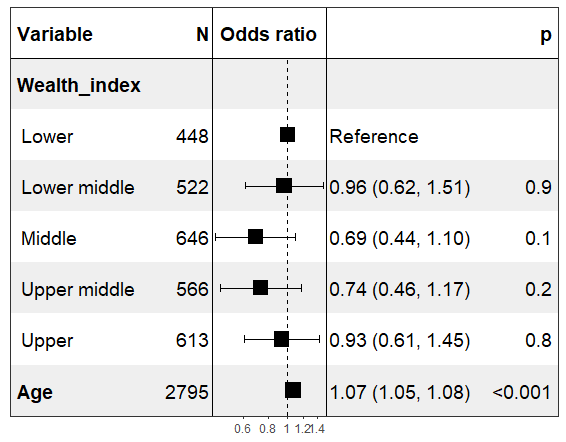 | 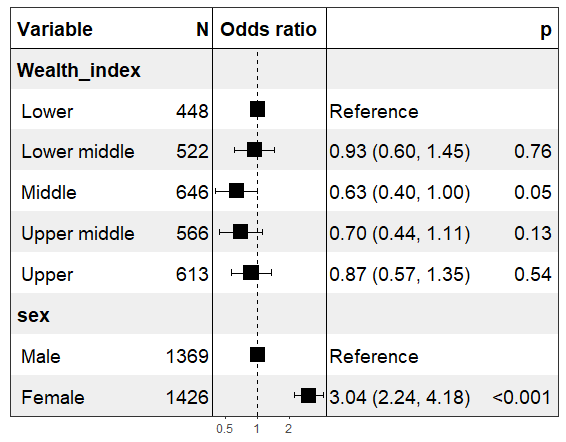 |
| **(d) Dementia~ Socioeconomic status + Education** | **(e) Dementia~ Socioeconomic status + Occupation** | **(f) Dementia~ Socioeconomic status +** Marital status |
| 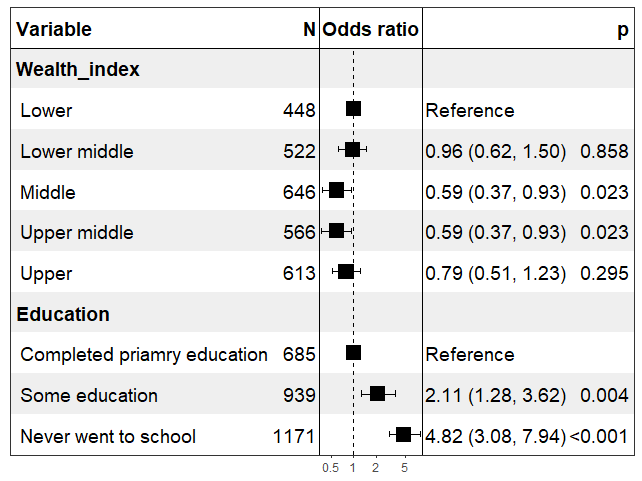 | 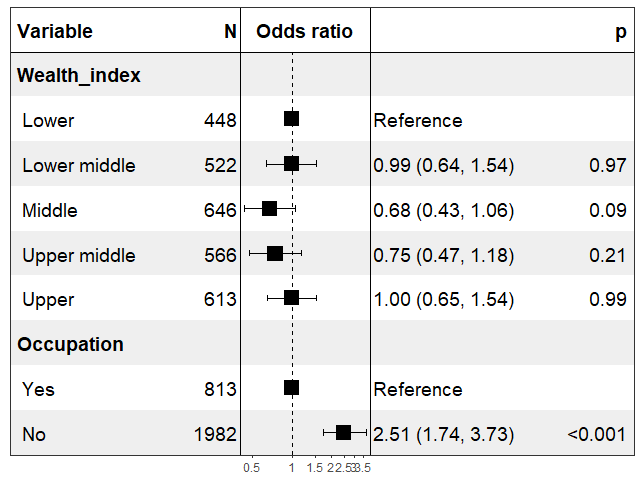 | 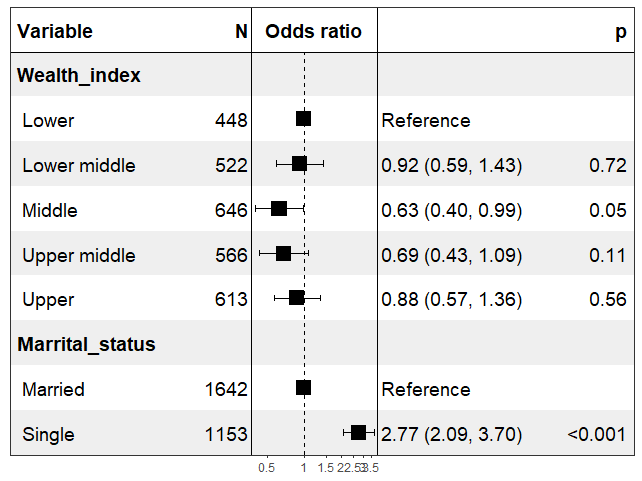 |
| **(g) Dementia~ Socioeconomic status +Type of community** |  |  |
| 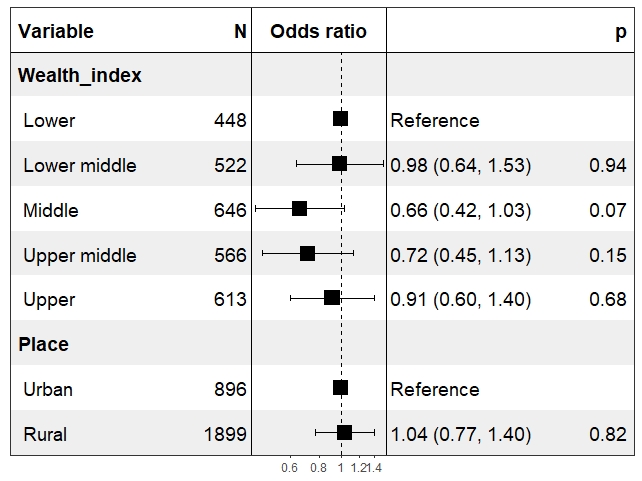 |  |  |

***Multivariable analysis: Stratified by sex and division***

**Figure S10. Factors associated with and without dementia stratified by sex**

| **(a) Female** | **(b) Male** |
| --- | --- |
| 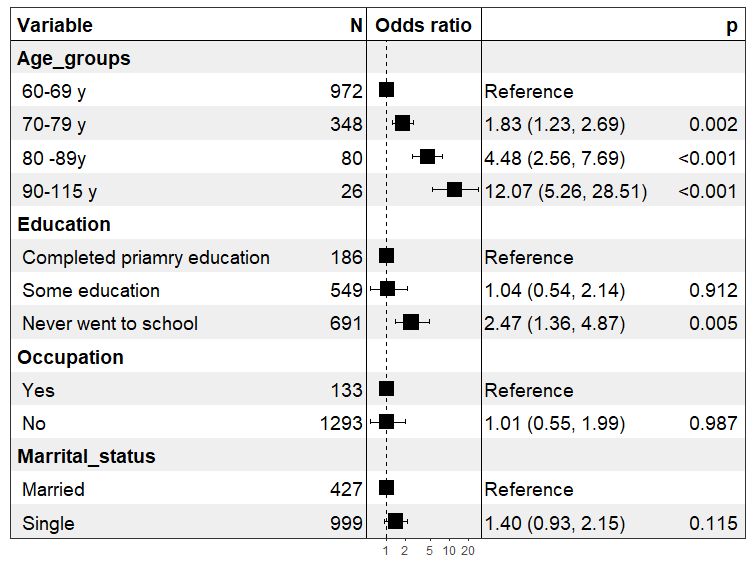 | 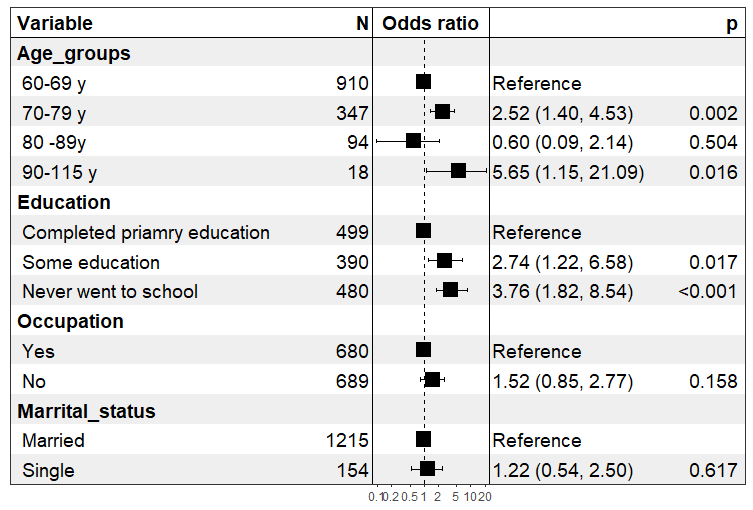 |

**Figure S11.** Factors associated with and without dementia stratified by division

| **(a) Chattogram** | **(b) Barisal** | **(c) Khulna** | |
| --- | --- | --- | --- |
| 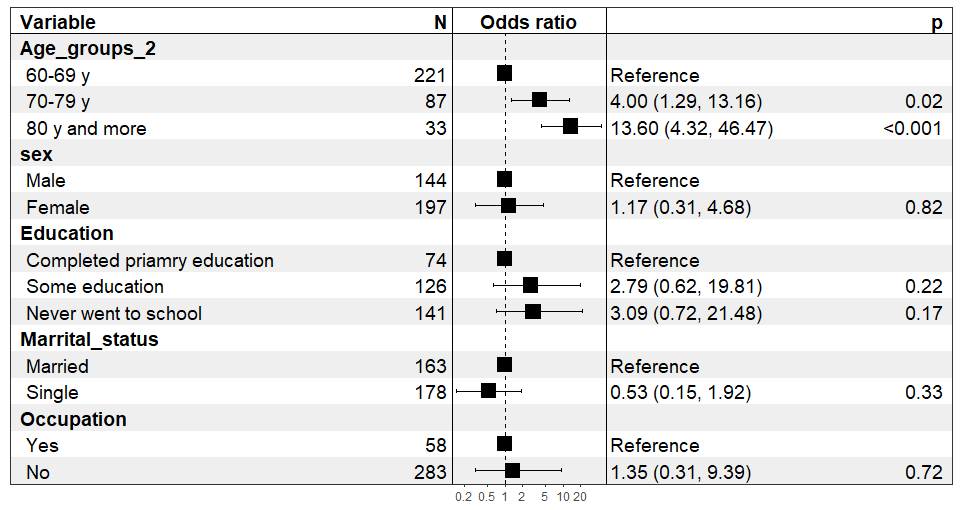 | 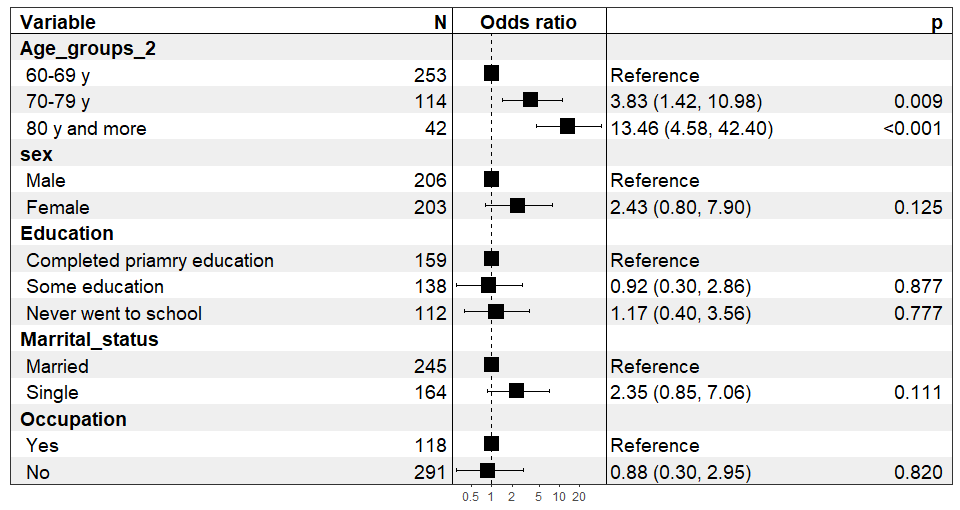 | 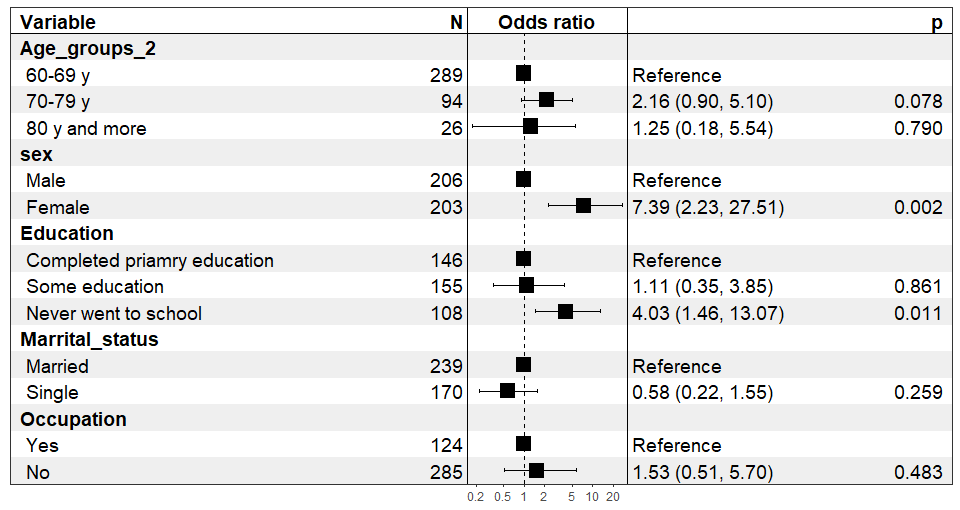 | |
| **(d) Sylhet^*^** | **(e) Rajshahi** | **(f) Rangpur** | |
| 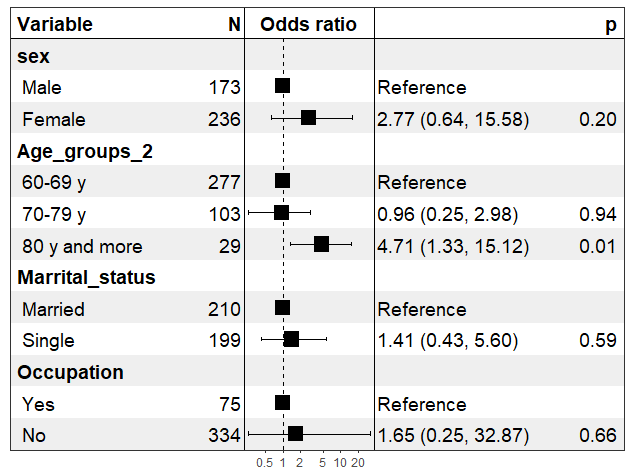 | 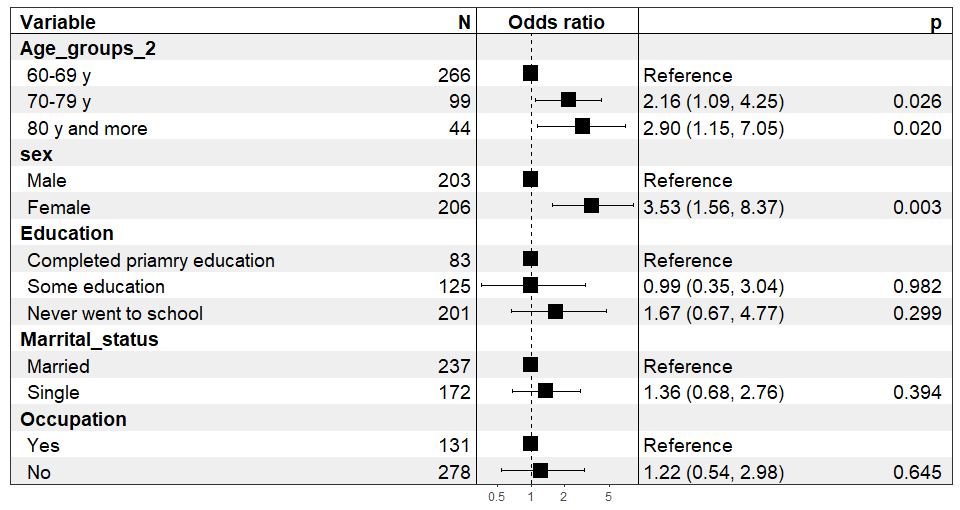 | 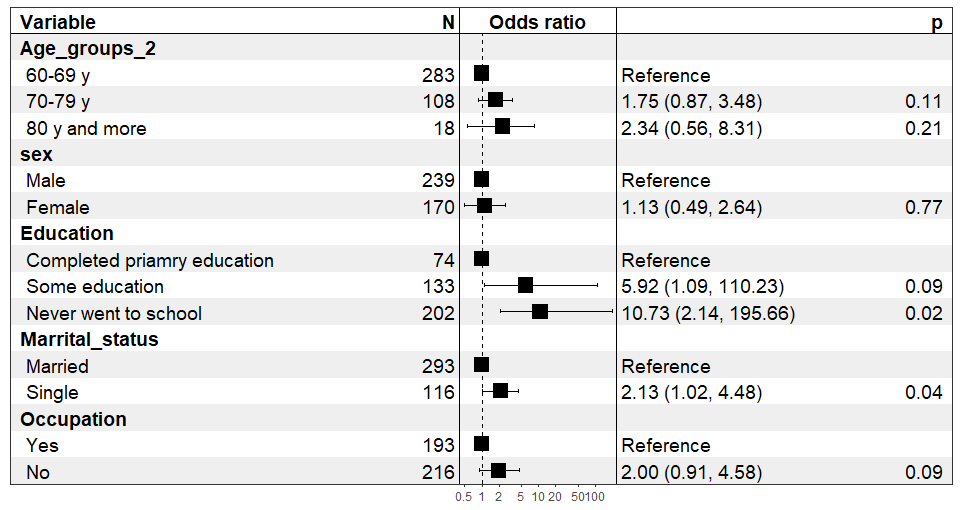 | |
| **(g) Dhaka^*^** |  |  | |
| 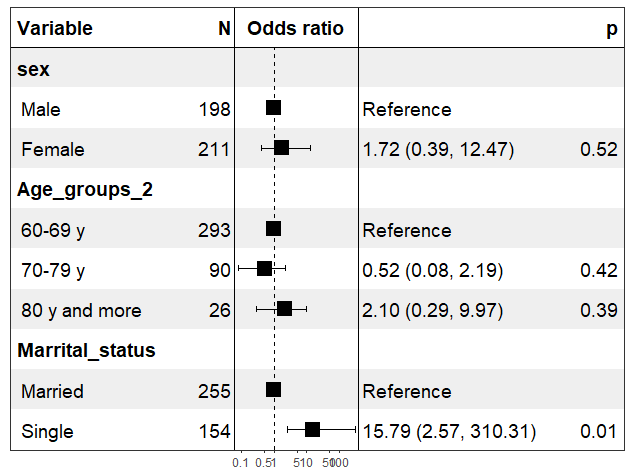 |  | | |
| *In Dhaka division, education and occupation variables excluded from model as dementia cases not found; In Sylhet division, education variable excluded from model as dementia cases not found | | |  |

**Multivariable analysis (Age adjusted as continuous variables)**

**Figure S12.** Factors associated with and without dementia of older people (Adjusted odds ratio with 95% CI)


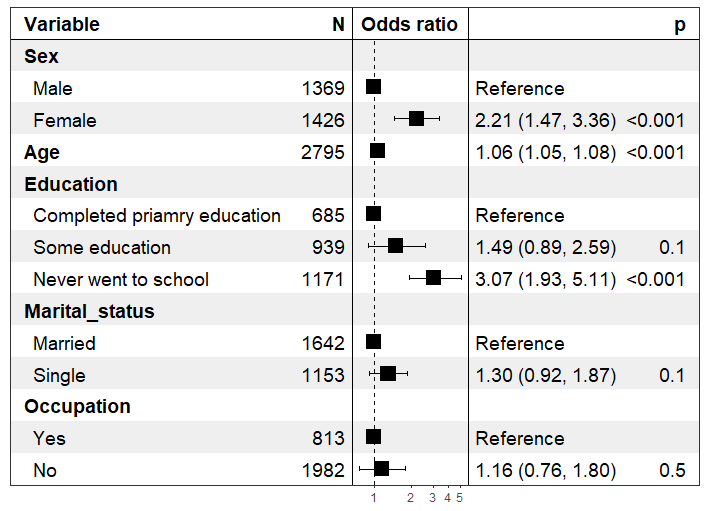


**Table S3**. Interaction analysis of sex and education with and without dementia (Logistic regression model^*^)

| Dementia | Adjusted odds Ratio | 95% CI | P-value |
| --- | --- | --- | --- |
| *Sex × Education level* |  |  |  |
| Male and completed primary education | Ref. |  |  |
| Male and some education | 2.62 | 1.14-5.99 | 0.02^**^ |
| Male and never went to school | 3.62 | 1.69-7.75 | P<0.001^***^ |
| Female and completed primary education | 3.57 | 1.42-9.00 | 0.01^**^ |
| Female and some education | 3.69 | 1.69-8.05 | P<0.001^***^ |
| Female and never went to school | 8.75 | 4.19-18.24 | P<0.001^***^ |

^*^Model adjusted with age, marital status, and occupation; Statistical significance at P<0.05^**^, P<0.001^***^

1. [↑](#footnote-ref-1)
